# Long-term hematopoietic stem cells trigger quiescence in *Leishmania* parasites

**DOI:** 10.1371/journal.ppat.1012181

**Published:** 2024-04-24

**Authors:** Laura Dirkx, Sara I. Van Acker, Yasmine Nicolaes, João Luís Reis Cunha, Rokaya Ahmad, Rik Hendrickx, Ben Caljon, Hideo Imamura, Didier G. Ebo, Daniel C. Jeffares, Yann G.-J. Sterckx, Louis Maes, Sarah Hendrickx, Guy Caljon

**Affiliations:** 1 Laboratory of Microbiology, Parasitology and Hygiene (LMPH), Infla-Med Centre of Excellence, University of Antwerp, Antwerp, Belgium; 2 York Biomedical Research Institute and Department of Biology, University of York, York, United Kingdom; 3 Brussels Interuniversity Genomics High Throughput core (BRIGHTcore) platform, Vrije Universiteit Brussel (VUB), Universitair Ziekenhuis Brussel (UZ Brussel), Brussels, Belgium; 4 Department of Immunology–Allergology–Rheumatology, Faculty of Medicine and Health Science, Infla-Med Centre of Excellence, University of Antwerp, Antwerp University Hospital, Antwerp, Belgium; 5 Laboratory of Medical Biochemistry (LMB), Infla-Med Centre of Excellence, University of Antwerp, Antwerp, Belgium; INRS - Institut Armand Frappier, CANADA

## Abstract

Addressing the challenges of quiescence and post-treatment relapse is of utmost importance in the microbiology field. This study shows that *Leishmania infantum* and *L. donovani* parasites rapidly enter into quiescence after an estimated 2–3 divisions in both human and mouse bone marrow stem cells. Interestingly, this behavior is not observed in macrophages, which are the primary host cells of the *Leishmania* parasite. Transcriptional comparison of the quiescent and non-quiescent metabolic states confirmed the overall decrease of gene expression as a hallmark of quiescence. Quiescent amastigotes display a reduced size and signs of a rapid evolutionary adaptation response with genetic alterations. Our study provides further evidence that this quiescent state significantly enhances resistance to treatment. Moreover, transitioning through quiescence is highly compatible with sand fly transmission and increases the potential of parasites to infect cells. Collectively, this work identified stem cells in the bone marrow as a niche where *Leishmania* quiescence occurs, with important implications for antiparasitic treatment and acquisition of virulence traits.

## Author summary

Quiescence and post-treatment relapse are crucial aspects of treatment failure across the microbiology field. This study shows that *Leishmania infantum* and *L. donovani* parasites rapidly enter into quiescence in both human and mouse bone marrow stem cells, but not in macrophages. Besides a reduced size, quiescent amastigotes show signs of a rapid evolutionary adaptation with notable genetic alterations. Transitioning through a quiescent state allows escape from treatment, efficient transmission by sand flies and the acquisition of an increased cellular infectivity. Transcriptional profiling of quiescent and non-quiescent parasites isolated from the stem cell niche confirmed a generalized transcriptional downregulation as a hallmark of quiescence.

## Introduction

Visceral leishmaniasis (VL) is a lethal neglected tropical disease caused by the obligate intracellular protozoan *Leishmania* [1,2] and transmitted through the bites of infected female phlebotomine sand flies [3,4]. *Leishmania* parasites alternate between two main morphological forms during their life cycle: a long flagellated extracellular promastigote within the sand fly and a non-flagellated obligate intracellular amastigote in the vertebrate host that resides within the monocyte-derived cells of the liver, spleen and bone marrow (BM) and eventually causes life-threatening complications [5–8].

The current antileishmanial drugs have many disadvantages and post-treatment relapse rates are increasing [9]. In many instances, relapse does not relate to reinfection, drug quality, drug exposure or resistance [10], but is rather due to persistence for which mechanistic information is lacking. Persistent infections can occur in a variety of host sanctuary tissues or cellular niches, such as hepatocytes (*Plasmodium vivax*), skeletal muscle and neurons (*Toxoplasma gondii*), adipose tissue (*Trypanosoma brucei* and *T. cruzi*) and the BM (*Mycobacterium tuberculosis*) [11–15]. Long-term hematopoietic stem cells (LT-HSC) in the BM were identified as a relapse niche for VL infection. LT-HSC become readily infected with extreme parasite burdens accompanied with low reactive oxygen species (ROS) and nitric oxide (NO) levels and a specific Stemleish transcriptional profile [16]. A recent dual-scRNA-seq analysis in a chronic *L. donovani* infection model corroborated the proportional importance of HSC, identifying them as the main parasitized cell type in the bone marrow [17].

Besides persistence linked to cellular niches, treatment failure can also be associated with the adaptive behavior of parasites. In response to stress, certain microorganisms employ so-called quiescence to increase their chances of survival [18]. The quiescent state is characterized by a lowered metabolic activity and renders a microorganism tolerant to antibiotics at the expense of becoming non-proliferative [19,20]. Hence quiescent cells are phenotypic variants of the wildtype, and their dormancy can be reversed when stressors are alleviated. Given its discernible clinical implications, microbial quiescence has gained considerable interest and has been the subject of intense research for certain pathogens, especially bacteria [19–23]. In contrast, quiescence in *Leishmania* has only recently been discovered and its role in drug tolerance, infection relapse and general parasite biology remains poorly understood. A recent study demonstrated that *Leishmania* quiescence can be induced by various triggers (*e*.*g*. antimonial drug pressure or stationary phase growth [24]). Furthermore, transcriptomic and metabolomic analyses of quiescent stages corroborated an overall downregulation of biosynthetic processes as a hallmark of quiescence [24]. Despite these important insights, the molecular determinants orchestrating the phenotypic transition to the quiescent state in *Leishmania* remain thus far unknown.

The present study started with the observation that visceral *Leishmania* amastigotes inside LT-HSC rapidly enter a quiescent state. Although the induction is unrelated to drug pressure, we demonstrate that these quiescent parasites benefit from an enhanced survival of antileishmanial treatment. To better understand the molecular basis underlying amastigote quiescence, we performed an unbiased total RNAseq, revealing an overall decreased gene expression as a hallmark of parasitic quiescence in the LT-HSC niche. In addition, we show that transitioning through a quiescent state has a profound impact on parasite infectivity and transmissibility. The results provide important information on the *in situ* acquisition of quiescence and its downstream effects on parasite biology (survival under drug pressure, infection, and transmissibility).

## Results

### 1) *Leishmania* infection of mouse and human stem cells triggers amastigote quiescence

**Fig 1:**
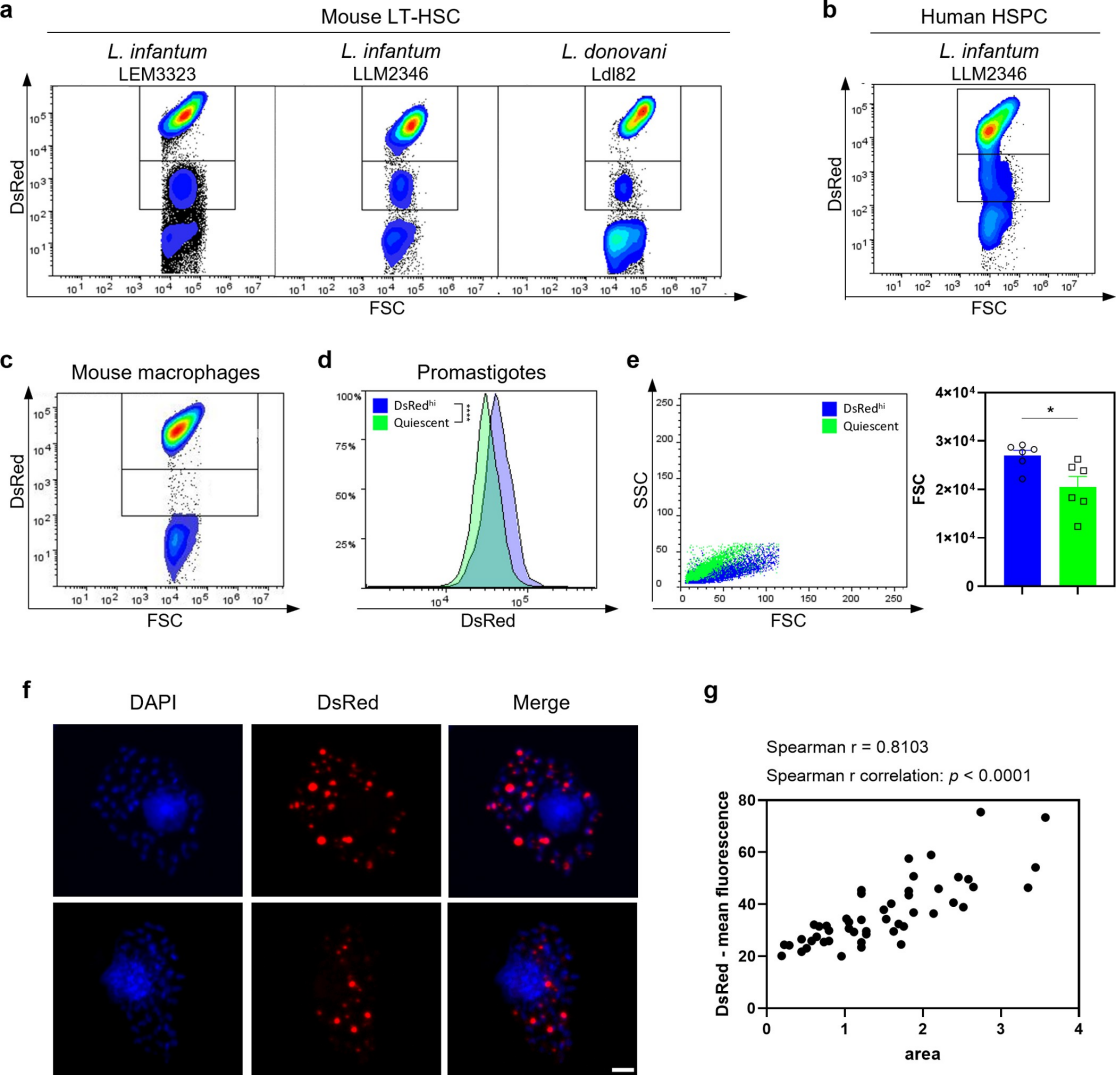
*Leishmania* infection of mouse LT-HSC and human HSPC triggers amastigote quiescence. **(a)** Amastigotes recovered from infected mouse LT-HSC and measured via flow cytometry. Cells in the left panel were infected with *L. infantum* LEM3323 WT^PpyRE9/DsRed^, middle panel with *L. infantum* clinical isolate LLM2346 WT^PpyRE9/DsRed^, right panel with *L. donovani* Ldl82 WT^PpyRE9/DsRed^. **(b)** Amastigotes recovered from *L. infantum* LLM2346 WT^PpyRE9/DsRed^ infected human HSPC. **(c)** BM derived macrophages were infected with *L. infantum* (LEM3323 WT^PpyRE9/DsRed^) and intracellular amastigotes were isolated and measured by flow cytometry. **(d)** DsRed expression measured by flow cytometry after promastigote back-transformation of DsRed^hi^ and DsRed^lo^ (*i*.*e*. quiescent) amastigotes recovered from LT-HSC. **(e)** *L. infantum* LEM3323 WT^PpyRE9/DsRed^ amastigotes recovered from infected mouse LT-HSC and measured via flow cytometry, back-gated on SSC versus FSC. Mann-Whitney test, *p < 0.05, six independent repeats. **(f)** Sorted mouse LT-HSC were infected with *L. infantum* (LEM3323 WT^PpyRE9/DsRed^) and processed for microscopy. DAPI (blue), amastigotes (red). Scale bar = 10 μm. **(g)** Analysis of microscopy images of (f) in the FIJI software, comparing the expression level of DsRed to its respective size.

Serendipitously we discovered by flow cytometry that already after 24 hours of *Leishmania* infection in LT-HSC, there is a presence of two distinct DsRed^+^ amastigote populations (DsRed^hi^ and DsRed^lo^) suggesting different metabolic states (**Fig 1A**) in the *dsRed*-transformed parasite line. Both populations remain at relatively constant proportions from 24 hours post infection (hpi) onwards. The decreased DsRed signal indicates reduced expression from the 18S rDNA locus, previously reported as an indicator of entry into a quiescent state [25]. *In situ* amastigote quiescence was shown to occur independently of strain (*L. infantum* LEM3323 or clinical isolate LLM2346) or species (*L. infantum* and *L. donovani*) (**Fig 1A**) and was recorded in both mouse LT-HSC and human hematopoietic stem and progenitor cells (HSPC) (**Fig 1B**). In contrast, amastigotes purified from infected macrophages cluster in one homogenous DsRed^hi^ population (**Fig 1C**). Promastigote back-transformation was used to confirm viability of sorted DsRed^lo^ parasites, the capacity to regain proliferative capacity and stability of the DsRed^lo^ phenotype. DsRed expression in the quiescent state remained lowered after transformation into the promastigote form (**Fig 1D**), which was also confirmed for derived monoclonal lines. Quiescent parasite cultures lost DsRed-expression after promastigote back-transformation with a frequency between 1.96% (1/51 clones) and 4.76% (2/42 clones) (**S1 Fig**), suggesting that parasites undergo a rapid evolutionary adaptation response and genetic rearrangements upon entry and exit from quiescence. Loss of the *dsRed* gene was demonstrated at RNA and DNA level by qPCR (**S1B Fig**). In contrast, no clones derived from DsRed^hi^ parasites lost the *dsRed* construct. Interestingly, quiescent amastigotes exhibit a significantly reduced size compared to DsRed^hi^ amastigotes (**Fig 1E**). Confocal fluorescence microscopy with z-stacking confirmed heterogenous DsRed signals and variable amastigote size in the LT-HSC (**Fig 1F and 1G**). This was corroborated by flow cytometry, as the mean fluorescence intensity of the DsRed signal increased with the apparent amastigote size (forward scatter–FSC, **Figs 1E, S2A and S2B**), illustrating that acquisition of quiescence is associated with several cellular changes.

### 2) Amastigotes enter quiescence following *in situ* proliferation in mouse and human stem cells

**Fig 2:**
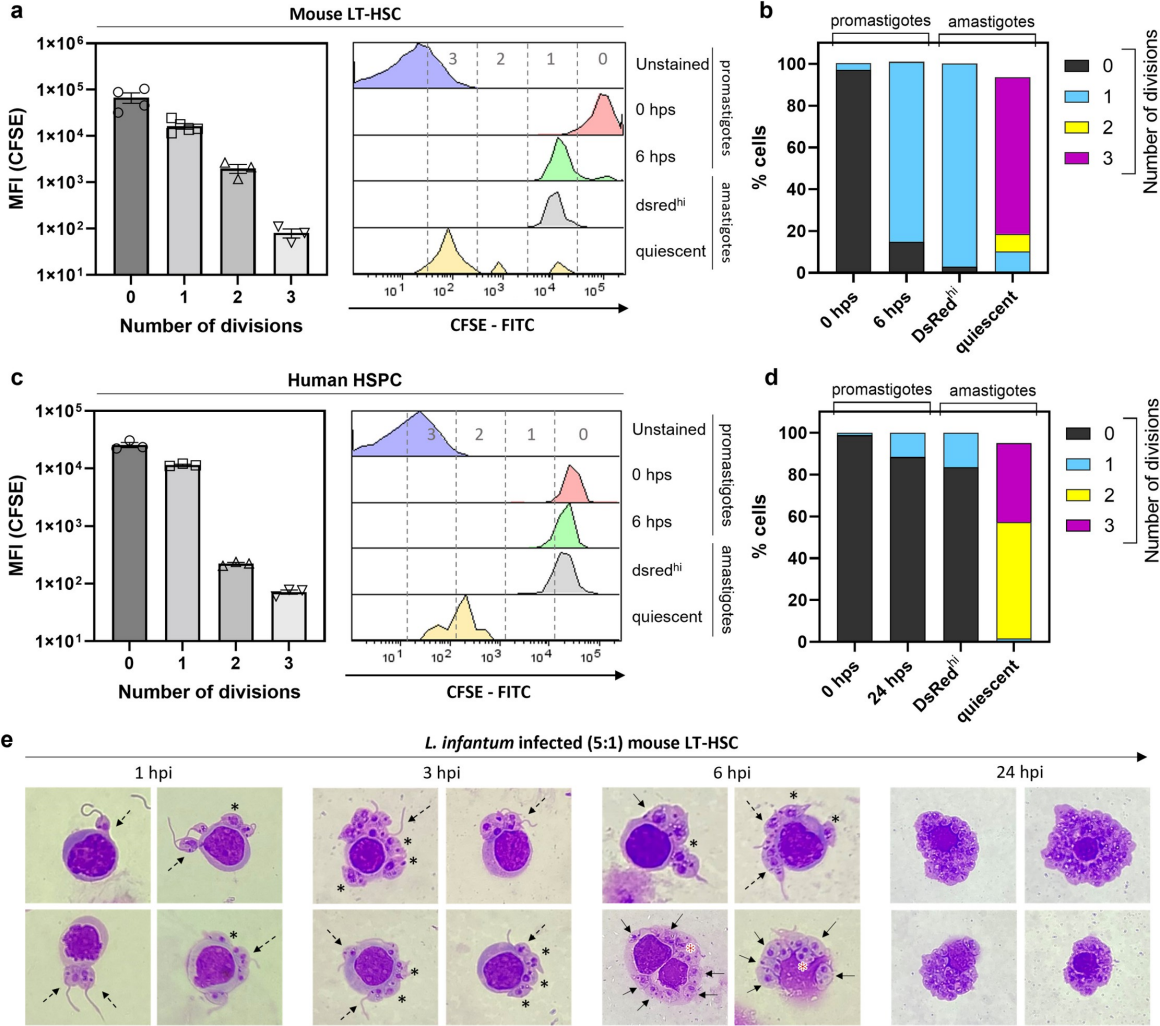
Number of divisions associated with quiescence in amastigotes from mouse LT-HSC and human HSPC. **(a)** Number of divisions as calculated by CFSE staining and defined by curve-fitting of the cellular CFSE-intensity using the Proliferation Analysis tool of FlowLogic (left panel). Controls are unstained and CFSE *L. infantum* (LEM3323 WT^PpyRE9/DsRed^) promastigote cultures. Sorted mouse LT-HSC were infected with CFSE labelled *L. infantum* promastigotes and amastigotes were recovered after 6 hours of co-incubation (right panel). **(b)** Percentage of cells in each division range from (a). Results are based on three independent repeats. **(c)** Number of divisions as calculated by CFSE staining using the Proliferation Analysis tool (left panel). Controls include unstained and CFSE-stained *L. infantum* (LLM2346 WT^PpyRE9/DsRed^) promastigote cultures. Sorted human HSPC were infected with CFSE labelled *L. infantum* promastigotes and amastigotes were recovered after 24 hours of co-incubation (right panel). **(d)** Percentage of cells in each division range from (c). Results are based on three independent repeats. **(e)** Early infection of *L. infantum* (LEM3323 WT^PpyRE9/DsRed^) in mouse LT-HSC at 1, 3, 6, and 24 hours post infection visualised by Giemsa staining. Amastigotes (arrow), promastigotes (dotted arrow), promastigotes transitioning to amastigotes, already without flagellum (asterisk), dividing amastigotes (red asterisk).

To uncover why LT-HSC trigger the rapid development of quiescent amastigotes, a CFSE labelling of *L. infantum* LEM3323 promastigotes was performed to assess the number of parasite divisions before entering into quiescence (*i*.*e*. acquire a DsRed^lo^ phenotype). The number of divisions was calculated based on curve-fitting of the cellular CFSE-intensity using the Proliferation Analysis tool of FlowLogic. The 0 hps (hours post staining) peak was considered as generation 0 and an unstained sample as baseline (**Fig 2A, left panel**). The CFSE profile at time 0 has negligible differences in initial parasite size, and most are metacyclic (FSC^lo^) with evenly distributed CFSE signal (**S2C Fig**). CFSE versus FSC profile does not show a correlation between CFSE intensity and event size (**S2D Fig**). After 6 hours of co-incubation, the DsRed^hi^ amastigote fraction divided about 1 time compared to the control (0 hours), which was comparable to promastigote proliferation in 6-hour *in vitro* cultures. In contrast, the amastigote fraction that would eventually acquire a DsRed^lo^ phenotype displayed a more diverse pattern, ranging between 1, 2 and 3 *in situ* divisions (**Fig 2A**). The highest proportion of amastigotes underwent 3 divisions to enter into quiescence (**Fig 2B**). These data indicate that the high proliferation rate in LT-HSC is associated with quiescence. To confirm clinical relevance of these findings, amastigote quiescence was tested in human HSPC using a recent *L. infantum* clinical isolate (LLM2346 WT^PpyRE9/DsRed^). In **Fig 2C and 2D**, CFSE labelling of promastigotes was again performed to assess the number of divisions. After 24 hours of co-incubation, the DsRed^hi^ fraction divided about 0–1 time compared to the control (0 hours), which was comparable to the promastigote culture after 24 hours. The amastigote fraction that ultimately becomes quiescent divided about 1–3 times (**Fig 2D**). The observed difference in division rate between LEM3323 and LLM2346 can be linked to intrinsic growth rate differences (**S3A and S3B Fig**). Collectively these data show that *Leishmania* quiescence arises mostly after 2–3 divisions in mouse and human stem cells. From **Fig 2E** it is apparent that transitioning promastigotes (asterisk) can already be detected as early as 1 hpi. Moreover, 6 hpi, intracellularly dividing amastigotes can be already clearly observed (red asterisk). By 24 hpi, the cell contains large numbers of amastigotes. These data unambiguously show that promastigotes rapidly convert into proliferating amastigotes in the HSC niche. This further documents the peculiar interaction of *Leishmania* with this specific cell type.

### 3) *In situ* quiescent amastigotes undergo vast transcriptional changes

To unravel the molecular basis for quiescence in LT-HSC amastigotes, unbiased total RNA sequencing was performed on three independent samples of 10,000 DsRed^lo^ (quiescent) and DsRed^hi^ (non-quiescent) amastigotes that were isolated and flow sorted from mouse LT-HSC in three independent infection experiments. Principal component analyses (**S4A and S4B Fig**) revealed distant profiles of the quiescent and DsRed^hi^ samples, supporting the observed difference between both parasite phenotypes. Consistent with a previous quiescence study **(24)**, ribosomal genes were strongly downregulated. The ribosomal genes (194 genes) and transfer RNA (37 genes) were removed for the downstream differential expression analysis. The total number of reads and median mapped read length were much lower in quiescent samples as compared to DsRed^hi^ samples (**S1 Data**). Strongly reduced transcript levels were confirmed by RT-dPCR on a set of 18 genes, indicating an average 9.3-fold decrease (**S4C Fig**). The used default mapping conditions (MAPQ 10 and no read length limit) were discovered unsuitable due to a very high proportion of fragmented reads in quiescent samples resulting in stochastic RNA read counting. Kraken2 read classification did not indicate any significant contaminations from bacteria, human, or mouse. Based on these observations, very strict criteria (MAPQ 60 and mapped read length greater than 90 bases) were implemented and only 167 nuclear genes with high-confidence reads were identified in the three independent quiescent samples (**S1 Data**). These genes were enriched in GO biological processes related to microtubule-based movement and cholesterol/ergosterol biosynthetic processes (**S4D Fig and S1 Data**). While a generalized reduction of transcript levels was evident, no obvious cellular stress response based on heat shock protein expression (only LINF_360027200 –Hsp60 was detected in the three quiescent samples) and no potential positive markers or drivers of quiescence could be identified.

### 4) Transition through *in situ* quiescence enhances parasite survival, infectivity, and transmission potential

**Fig 3:**
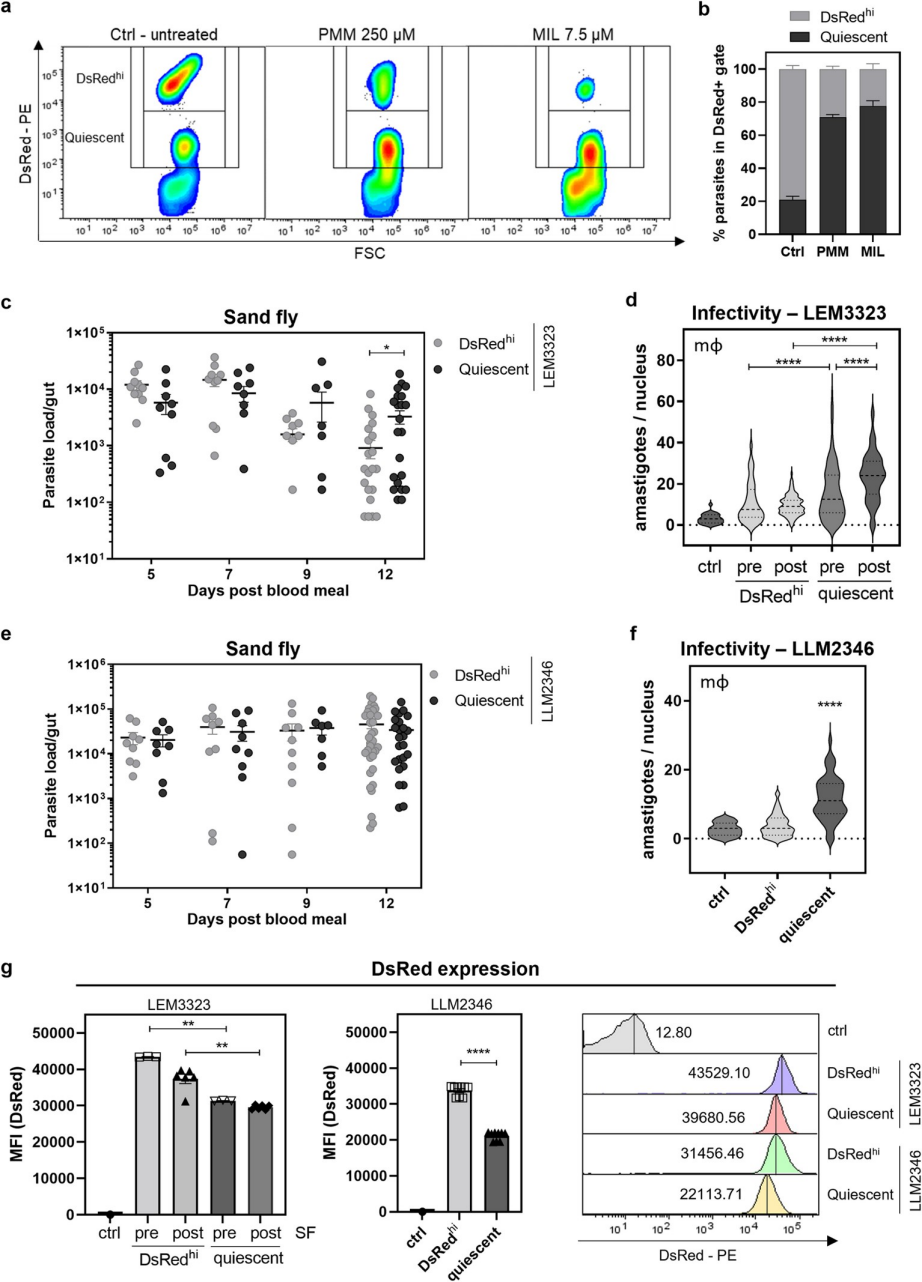
Phenotypic characteristics of quiescent amastigotes from LT-HSC. **(a-b)** Sorted LT-HSC were infected with *L. infantum* (LEM3323 WT^PpyRE9/DsRed^) for 24 hours followed by treatment with 250 μM PMM or 7.5 μM MIL for 72 hours. To compare pre- and post-treatment distribution of quiescent parasites, amastigotes were isolated and remeasured on the FACSMelody. **(c)** Sand flies were infected by *L. infantum* LEM3323 promastigotes recovered from DsRed^hi^ and quiescent amastigotes in mouse LT-HSC. The parasite load in the gut was assessed at days 5, 7, 9 and 12 after infection (blood meal). Sand fly infections were repeated three independent times. Unpaired *t* test, 10 < *n* < 30, *p <0.05. **(d)** *L. infantum* LEM3323 promastigotes of a control culture and promastigotes recovered from DsRed^hi^ and quiescent parasites in mouse LT-HSC, pre- and post-sand fly passage (pre- and post-SF) were co-incubated with peritoneal macrophages for 96 hours and infectivity was assessed with Giemsa staining. Cultures were visually confirmed to contain > 90% metacyclics and normalized by counting to expose macrophages at a MOI of 5 (**S6 Fig**). The original promastigote culture was used as a control and infectivity was found to be significantly lower than those of pre-SF DsRed^hi^ (*p* <0.01), post-SF DsRed^hi^ (*p* <0.05), and pre- and post-SF quiescent parasites (*p* <0.0001). Ordinary one-way ANOVA, 30 < *n* < 130, ****p <0.0001. % infected cells and number of parasites per 100 macrophages are included in **S1 Table**. **(e)** Sand flies were infected with *L. infantum* LLM2346 promastigotes, recovered from DsRed^hi^ and quiescent amastigotes in human HSPC. The parasite load in the gut was assessed at days 5, 7, 9 and 12 after the infectious blood meal. Sand fly infections were repeated three independent times. 10 < *n* < 34. **(f)** *L. infantum* LLM2346 promastigotes of DsRed^hi^ and quiescent recovered promastigotes from human HSPC were co-incubated with peritoneal macrophages for 96 hours and infectivity was assessed with Giemsa. Mann-Whitney test, *n* = 100, ****p <0.0001. **(g)** DsRed expression of *L. infantum* LEM3323 DsRed^hi^ and quiescent promastigotes recovered from mouse LT-HSC before or after passage through the sand fly, and *L. infantum* LLM2346 DsRed^hi^ and quiescent promastigotes recovered from human HSPC. Mann-Whitney test, **p <0.01. All experiments are expressed as mean ± SEM.

Next, we wondered whether adoption of a quiescent state would have an impact on various aspects of parasite biology. To investigate whether amastigote quiescence in LT-HSC is linked to survival of treatment and consequent occurrence of relapse, sorted/infected cells were treated with 250 μM paromomycin (PMM) or 7.5 μM miltefosine (MIL) for 72 hours before purifying the remaining amastigotes and determining the distribution of quiescent parasites based on DsRed fluorescence by flow cytometry. Drug treatment was found to primarily affect DsRed^hi^ parasites, increasing the proportion of quiescent amastigotes (**Fig 3A and 3B**). In macrophages, drug pressure slightly increased the number of DsRed^lo^/quiescent parasites (**S5 Fig**), albeit resulting in much lower proportions than those observed in LT-HSC. Moreover, this study demonstrates that quiescence occurs naturally in LT-HSC in the absence of drug pressure.

To assess whether going through a reversible quiescent state influences subsequent transmission, sand flies were infected with promastigotes derived from DsRed^hi^ and quiescent amastigotes. In **Fig 3C**, parasite load in the gut was compared at different time points, with parasites having transitioned through a quiescent state showing a slightly enhanced sand fly infectivity (*p* <0.05 at day 12). A similar analysis was performed for parasites derived from human HSPC, where infectivity in the sand fly vector remained the same (**Fig 3E**).

As shown before sand fly passage, quiescent parasites post-sand fly passage showed a lower DsRed signal (**Fig 3G**), indicating that some quiescence-associated phenotypic changes are stable after transmission. Infectivity was evaluated by co-incubation with peritoneal macrophages for 96 hours. Interestingly, promastigotes derived from quiescent (DsRed^lo^) amastigotes had a significantly higher infectivity compared to their DsRed^hi^ counterparts (*p* <0.0001) or the original promastigote culture before LT-HSC passage (*p* <0.0001). This difference in infectivity was even more pronounced after sand fly passage (**Fig 3D**). The infectivity in macrophages was also observed to be significantly higher for promastigotes derived from the quiescent strain recovered from human HSPC (*p* <0.0001) than for DsRed^hi^ promastigotes (**Fig 3F**). The *in vitro* growth curves of *L. infantum* LEM3323 and LLM2346 promastigotes comparing quiescent and DsRed^hi^ phenotypes show no biologically significant differences, suggesting that growth rate is not a determining factor contributing to the observed higher infectivity (**S3A and S3B Fig**). These results highlight that a transition through quiescence not only affects treatment but also significantly influences important life cycle features such as infectivity and transmissibility.

### 5) *In vivo* relapse parasites share characteristics with parasites that transitioned through quiescence

**Fig 4:**
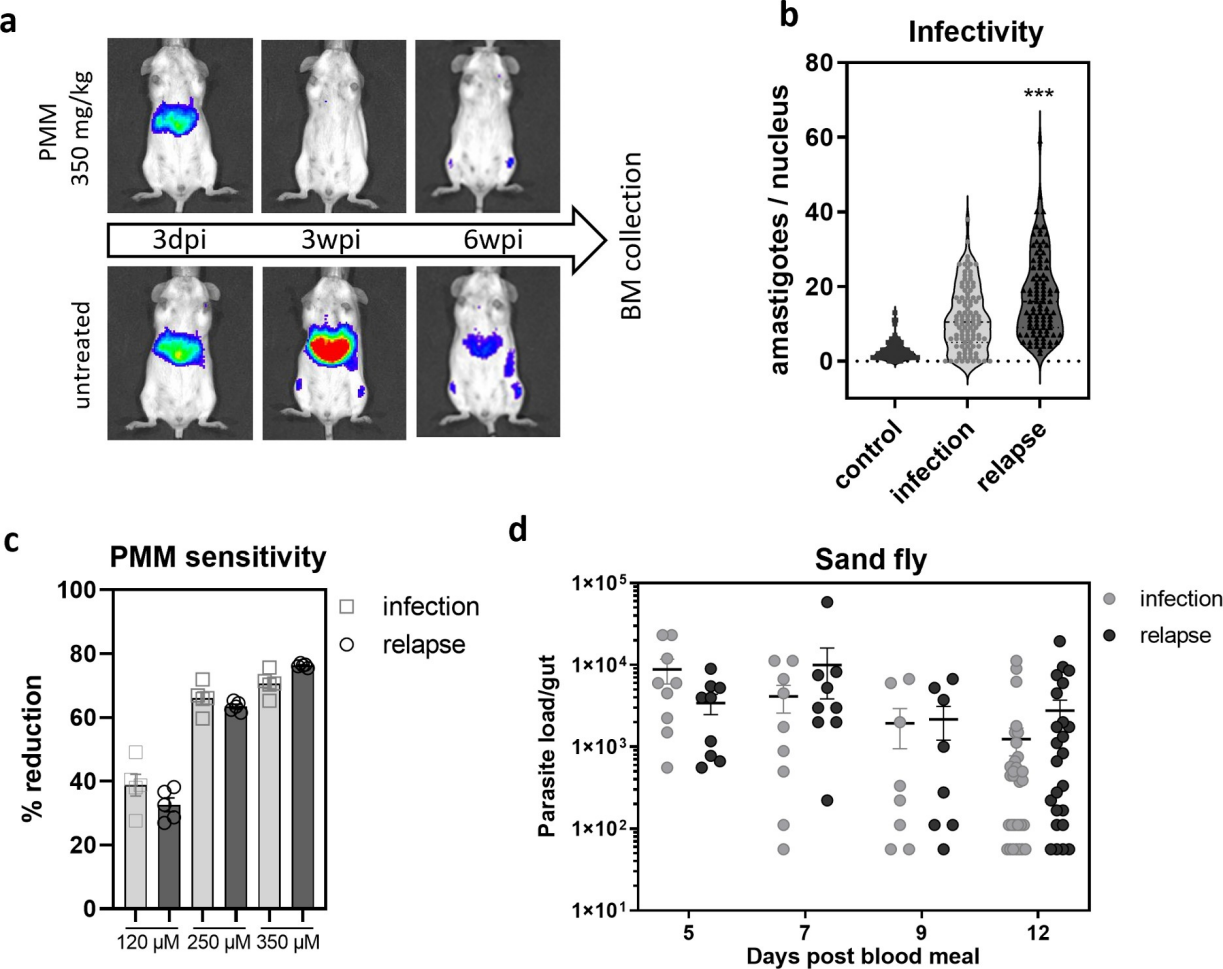
Phenotypic characteristics of promastigotes from relapsed BM. **(a)** BALB/c mice were infected with 10^8^ stationary phase promastigotes of *L. infantum* LEM3323 WT^PpyRE9/DsRed^. One group was treated with PMM 350 mg/kg per day (IP) for 5 consecutive days. Infection was followed up by BLI, BM was collected at 6 weeks post infection (wpi) from untreated and relapsed mice. **(b)** Stationary phase promastigotes recovered from relapsed BM (relapse) and untreated BM (infection) were co-incubated at a MOI of 5 with peritoneal macrophages for 96 hours and infectivity was assessed with Giemsa. Parasite cultures were counted and visually confirmed to contain > 90% metacyclics to exclude an infectivity bias based on the parasite culture (**S6 Fig**). Mann-Whitney test, *n* = 100, ***p <0.001. % infected cells and number of parasites per 100 macrophages are included in **S1 Table**. **(c)** *L. infantum* LEM3323 promastigotes recovered from relapsed BM (relapse) and untreated BM (infection) were co-incubated with peritoneal macrophages for 24 hours and treated with 120 μM, 250 μM or 350 μM PMM for 72 hours, infectivity was assessed with Giemsa. **(d)** Sand flies were infected by *L. infantum* LEM3323 promastigotes recovered from relapsed BM (relapse) and untreated BM (infection) and the parasite load in the gut of infected flies was assessed at days 5, 7, 9 and 12 after infection (blood meal). Sand fly infections were repeated three independent times. 10 < *n* < 32.

Using a previously described reproducible post-treatment relapse model [16], mice were sacrificed at 6 weeks post infection (4 weeks post PMM treatment) and BM was collected for promastigote back-transformation (**Fig 4A**). Infection of mouse peritoneal macrophages revealed an enhanced infectivity of relapse parasites (**Fig 4B**) as was found for quiescent parasites. Infection phenotypes can often be attributed to different levels of metacyclic parasites (FSC^lo^, [26]). To exclude such bias, flow cytometry analysis and visual inspection of the cultures were performed, revealing similar FSC/SSC profiles and visually confirming > 90% metacyclics (**S6 Fig**). Moreover, growth curves shown in **S3C Fig** demonstrate no discernable differences in promastigote growth over 10 consecutive days of culture. The percentage of reduction after *in vitro* PMM treatment of infected macrophages remained stable for relapse versus the parental parasites, demonstrating that these parasites did not acquire a drug resistant phenotype (**Fig 4C**). To check whether the parasites were still infective for sand flies, the gut parasite load was compared at different time points. No significant differences in sand fly parasite loads (**Fig 4D**) and *in vitro* promastigote growth (**S3C Fig**) were detected. The recorded high fitness of relapse parasites overlaps with the parasite phenotype after transition through a quiescent stage in LT-HSC.

## Discussion

Current resources for new drug development for VL are scarce. Despite the imminent need to combat the increasing relapse rates, little is known on the underlying cause and suitable methods to study this phenomenon. Notably, post-treatment relapse is mostly not due to reinfection, drug quality, drug exposure or drug-resistant parasites [10,27], but rather due to persistence of the pathogen. This persistence causing subclinical infections and subsequent relapse has been widely described across the microbiological spectrum [11–15,28–30]. Generally, two aspects can be the cause of this: pathogens residing in sanctuary sites or niches, or pathogens switching to a quiescent phenotype. The first allows pathogens to survive and escape treatment or immunity without genetic or phenotypic changes. The latter relies on phenotypic diversity, *e*.*g*. quiescent or dormant forms [31]. Based on our observations, treatment failure in VL most likely results from an intrinsic ability of the *Leishmania* parasite to benefit from both quiescence and the occupation of sanctuary sites. We have previously identified a relapse-prone cellular niche in the BM in which treatment is less effective [16]. Here, we formally link quiescent parasites residing in this particular sanctuary site to drug tolerance.

In literature there is no clear consensus on the definition of persistence or quiescence, and additional synonyms such as dormancy and latency are used interchangeably as conceptually related terms. More commonly, quiescence refers to genetically drug susceptible, dormant (non-growing or slow growing) organisms that survive exposure to a given cidal drug and have the capacity to regrow (or resuscitate and grow) under highly specific conditions [32]. Several external triggers may induce quiescence, such as host immunity, drug pressure and/or nutritional and energetic stress [21,33]. It is possible that the lowered production of ROS and NO in infected LT-HSC as documented in our previous work [16] creates a more hospitable environment for *Leishmania* survival and multiplication [34], and underly the specific host cell characteristics that support the emergence of parasitic quiescence. Investigation of the physiology of this phenotype *in vivo* is very challenging due to their scarcity and difficulty of detection. A review by Barrett *et al*. envisages several methods to study quiescence such as fluorescent probes, DNA replication probes, sorting of quiescent *vs* replicating cells, and several omics approaches [31]. Studies in various model organisms have been proposed to gain insights in the enigmatic basis of quiescence, such as transgenic fluorescent hypnozoites for *P. vivax* [35], multiple-stress model to study *M. tuberculosis* [36], and GFP reporter gene expression from an 18S rDNA locus in cutaneous leishmaniasis [25].

Quiescence in (cutaneous) leishmaniasis has been observed in several experimental systems since the first observation in 2015 by Kloehn *et al*. [37]. A form of quiescence has been described in axenic cultures of cutaneous *Leishmania* strains treated with antimonials or grown under stress conditions [24,25). A recent study on *L. mexicana* dermal granulomas describes a mosaic of metabolically active and semi-quiescent parasites during acute phases of infection, linking the phenotype to treatment failure [38]. Another study uses *in vivo* labeling with bromodeoxyuridine (BrdU) to visualize persistent slow-growing *L. major* parasites in macrophages in the skin [39]. To date, a knowledge gap exists for naturally elicited quiescent parasites recovered from a host cell niche, especially for VL. The present study provides unprecedented insights into quiescence of VL parasites in LT-HSC. The *Leishmania* amastigote stage had already been described as a less active state that may represent an adaptive response to a growth-restrictive intracellular microenvironment in granulomas [37,40]. Here, we compared amastigotes within the common macrophage host cell to those within the LT-HSC niche and found distinct differences. Within LT-HSC, quiescence occurred within 6 hours following an estimated 2–3 divisions that affect amastigote size and are linked to rapid genetic alterations as observed by the erosion of the DsRed expression cassette. This stably reduced expression of DsRed that is also present in the promastigote form is possibly linked to aneuploidy, with chromosomal copy number reduction corresponding to reduced transcript and protein levels [41]. This indicates that the high intracellular parasite proliferation rate in the stem cell is associated with the early emergence of quiescence.

To study the potential mechanisms underlying *Leishmania* quiescence, transcriptomic analyses were conducted on sorted DsRed^hi^ and DsRed^lo^ parasites by RT-dPCR and an unbiased total RNAseq. RT-dPCR on a set of 18 tested genes, revealed an order of magnitude lower expression (average 9.3-fold) in quiescent parasites as compared to DsRed^hi^ samples. RNAseq corroborated a much lower number of reads and median mapped read length in quiescent samples. We observed a high proportion of fragmented short reads of lower mapping quality in DsRed^lo^ samples and therefore implemented more strict mapping criteria to exclude the stochastic low-quality reads. As a result, reads for only 167 nuclear genes were detected in the three independent quiescent samples, which were enriched in GO biological processes related to microtubule-based movement and cholesterol/ergosterol biosynthetic processes. No obvious signs of a stress response with the expression of stress related HSPs were noted [42–44]. The overall downregulation of RNA, including ribosomal protein genes, is a signature of quiescence well described in literature [24,37,45,46], as ribosome biosynthesis is one of the most energy intensive processes in the cell and thus a measure of the metabolic state. Studies in *Plasmodium spp*. and *Toxoplasma gondii* persisters have shown that DNA replication, general transcription and protein synthesis are decreased [31]. Axenic amastigote forms of *L. mexicana* and *L. braziliensis* showed downregulated synthesis of ATP, ribosomal components, proteins and alterations in membrane lipids [25,40]. Mitochondrial gene expression has also been described to be reduced in an artificial model of quiescence [24]. In contrast to many other species, *Leishmania* does not have transcriptional regulation and as such is mainly compensated by post-transcriptional mechanisms [47]. Indeed, *Leishmania* is known for its extreme genomic and phenotypic variability, whereby a rapid shift in the gene repertoire is one of the key mechanisms for swift adaptation to changing environments [48], some of which have been associated with increased fitness in stress conditions or drug resistance [49]. Characterizing quiescent VL parasites recovered from LT-HSC showed an increased cellular infectivity and a high capacity to colonize the sand fly gut. Consistent with the quiescent phenotype, parasites recovered from the BM of relapsed mice, of which LT-HSC were previously shown to be the main parasitized cells [16], show an increased fitness with an elevated macrophage infectivity combined with a high sand fly infectivity. This suggests that the selected phenotype, typically associated with transition through a quiescent state, may pose an additional threat to leishmaniasis control programs. An increased infectivity associated with relapse of *L. donovani* infection has already been shown for miltefosine and antimonial treatment in the Indian subcontinent [10,50,51]. To prevent treatment failure, we advocate that the quiescent state of *Leishmania* should be considered in the early stages of the drug discovery process. Given its broad relevance across the microbial spectrum, further exploration of parasitic quiescence in the LT-HSC niche is warranted to identify potential liabilities for therapeutic intervention.

## Materials and methods

### Ethical statement

The use of laboratory rodents was carried out in strict accordance with all mandatory guidelines (EU directives, including the Revised Directive 2010/63/EU on the Protection of Animals used for Scientific Purposes that came into force on 01/01/2013, and the declaration of Helsinki in its latest version) and was approved by the Ethical Committee of the University of Antwerp, Belgium (UA-ECD 2019–04). Human bone marrow aspirate rest samples, obtained as a diagnostic sample without a written informed consent, were available for *in vitro* infection experiments following approval by the Committee of Medical Ethics of the Antwerp University Hospital (B3002021000027). In accordance with Article 20, §1 of the Belgian decree of 19 December 2008, body material that remains after a diagnostic examination or an intervention (residual material or residual tissue) can be used for scientific research. Patients or legal representatives have the right to refuse this at any time and can communicate this refusal to the responsible doctor or the medical director.

### *Leishmania* parasites

The *L. infantum* strains MHOM/FR/96/LEM3323 and MHOM/ES/2016/LLM2346 were kindly provided respectively by CNRL (Montpellier, France) and by WHOCC (Madrid, Spain), the latter being a recent clinical isolate. The *L. donovani* strain MHOM/ET/67/L82 was isolated from an Ethiopian VL patient. All were modified to express bioluminescent (PpyRE9) and fluorescent (DsRed) reporter genes integrated into the 18S rDNA locus (LEM3323 WT^PpyRE9/DsRed^, LLM2346 WT^PpyRE9/DsRed^ and Ldl82 WT^PpyRE9/DsRed^) [52,53]. Promastigotes were sub-cultured twice weekly at 25°C in hemoflagellate-modified minimal essential medium (HOMEM, Gibco), supplemented with 10% inactivated fetal calf serum (iFCS), 200 mM L-glutamine, 16.5 mM NaHCO_3_, 40 mg/L adenine, 3 mg/L folic acid, 2 mg/L D-biotin and 2.5 mg/L hemin. The number of passages was kept as low as possible to maintain parasite virulence.

### Laboratory animals and sand fly colony

Female BALB/c mice (6–8 weeks old) were purchased from Janvier (Genest-Saint-Isle, France) and accommodated in individually ventilated cages in SPF conditions. They were provided with food for laboratory rodents (Carfil, Arendonk, Belgium) and water *ad libitum*. Animals were subdivided in experimental groups based on simple randomization. Mice were kept in quarantine for at least 5 days before starting the experiment. Euthanasia was performed in CO_2_ chambers followed by cervical dislocation, and tissues were collected under aseptic conditions.

A *Lutzomyia longipalpis* sand fly colony was initiated with the kind help of NIH-NIAID (Prof. Shaden Kamhawi and Prof. Jesus Valenzuela) and maintained at the University of Antwerp under standard conditions (26°C, > 75% humidity, in the dark) with provision of a 30% glucose solution *ad libitum* [54]. For infection experiments, 3- to 5-day old females from generations 31 to 44 were used.

### Primary mouse cells

Mouse BM was collected from BALB/c mice using two distinct techniques, based on pilot studies comparing alternative methods in terms of yield and quality. For both techniques, mice were sacrificed, and hind legs aseptically removed. Isolated femurs and tibias were cleaned by removing soft tissue from the bone using 70% ethanol-soaked cloth and tweezers.

For the crushing technique, the protocol was adapted from Lo Celso and Scadden [55]. Briefly, bones were crushed with mortar and pestle in ammonium-chloride-potassium (ACK) buffer (0.15 M NH_4_Cl, 1.0 mM KHCO_3_, 0.1 mM Na_2_EDTA) for erythrocyte lysis. Single cell suspensions were obtained by filtering through MACS SmartStrainers (100 μm, Miltenyi Biotec), centrifuged at 500×*g* for 10 min (4°C) and resuspended in phosphate-buffered saline (PBS) + 0.2% bovine serum albumin (BSA). For efficient depletion of mature lineage-positive hematopoietic cells and to specifically isolate the preferred lineage-negative cells (*i*.*e*. undifferentiated progenitor cells), the Direct Lineage Cell Depletion Kit (Miltenyi Biotec) was employed according to manufacturer’s instructions. Following lineage depletion, cells were counted in PBS using a KOVA counting chamber and resuspended in PBS + 0.2% BSA buffer to 2×10^7^ cells/mL. Cells were kept on ice during all procedures.

The centrifugation method, adjusted from the protocol described by Amend *et al*. [56] and Dobson *et al*. [57], was used for subsequent macrophage and dendritic cell differentiation. Briefly, a 0.5 mL microcentrifuge tube was perforated at the bottom with a 21G needle and nested inside a 1.5 mL tube (both from Eppendorf). After collection of femurs and tibias, one proximal end (knee epiphysis) was cut-off and placed in the 0.5 mL tube. Nested tubes were centrifuged in a microcentrifuge at 10,000×*g* for 15 sec, resulting in a visible pellet in the 1.5 mL tube. This pellet was then resuspended in ACK buffer for erythrocyte lysis.

To obtain BM-derived macrophages (BMDM), cells were centrifuged at 500×*g* for 10 min at 4°C, resuspended in Roswell Park Memorial Institute (RPMI) medium (Gibco) and divided over Petri dishes (Starstedt) supplemented with BM medium [RPMI 1640 medium with 10% (v/v) iFCS, 1% non-essential amino acids (NEAA), 1% sodium pyruvate, 1% L-glutamine, 50 U/mL penicillin, 50 μg/mL streptomycin (all from Gibco) and 15% L929 supernatant with M-CSF]. Following a 6-day incubation at 37°C with 5% CO_2_, the macrophages were collected by replacing the BM medium with ice cold dissociation buffer [PBS with 1% 0.5 M ethylenediaminetetraacetic acid (EDTA) and 2% 1 M 4-(2-hydroxyethyl)-1-piperazine-ethanesulfonic acid (HEPES)]. After detachment, the macrophage cell suspension was centrifuged at 500×*g* for 10 min and resuspended in RPMI medium. Cells were seeded in a 96-well plate (3×10^4^ cells/well) or a 24-well plate (1×10^6^ cells/well) and incubated for 24 h at 37°C with 5% CO_2_ to allow adherence of the BMDMs.

Primary peritoneal macrophages were obtained from Swiss mice after inoculation of 1 mL 2% starch solution in PBS. Macrophages were seeded in a 96-well plate (6×10^4^ cells/well) and kept at 37°C and 5% CO_2_ to allow adhesion. After 48 hours, macrophages were infected as described below.

### Primary human BM cells

Human BM aspirate was obtained from the iliac crest using BD Vacutainer Plastic K3EDTA Tubes, initially collected for diagnostics, and delivered as residual sample. The BM was subjected to erythrocyte lysis twice using ACK buffer. Single cell suspensions were obtained by filtering through MACS SmartStrainers (100 μm, Miltenyi Biotec), centrifuged at 300×*g* for 10 min (4°C) and resuspended in PBS + 0.2% BSA. Cells were counted in PBS and diluted to 2×10^7^ cells/mL for flow cytometric analysis. Cells were kept on ice during these procedures.

### *In vitro* and *in vivo Leishmania* infections

Parasite density was assessed using a KOVA counting chamber. For *in vitro* infections, macrophages, LT-HSC and human hematopoietic stem and progenitor cells (HSPC) were co-cultured with stationary-phase promastigotes at a multiplicity of infection (MOI) of 5 for a minimum of 24h at 37°C with 5% CO_2_. Parasite cultures were inspected visually and measured by flow cytometry to confirm the presence of > 90% metacyclics, to exclude bias as a result of differences in metacyclogenesis in the cultures (**S6 Fig**). For post-passage infections (both after sand fly and *in vitro* HSC infections, *vide infra*), parasites were recovered in HOMEM medium at 25°C and checked daily for growth. Parasites were then transferred to a T25 flask in parallel and passaged once before determining the percentage of metacyclics using flow cytometry. For *in vivo* infection, stationary-phase parasites were centrifuged for 10 min at 4,000×*g* (25°C) and resuspended to 1×10^9^ parasites/mL in sterile RPMI medium. Mice were infected intravenously (i.v.) in the lateral tail vein with 1×10^8^ parasites in 100 μL of RPMI medium. Animals were monitored using *in vivo* bioluminescence imaging (BLI) at selected time points. Imaging was performed 3 min after intraperitoneal (i.p.) injection of 150 mg/kg D-Luciferin (Beetle Luciferin Potassium Salt, Promega) in the IVIS Spectrum In Vivo Imaging System under 2.5% isoflurane inhalation anesthesia using 15 min exposure. Images were analysed using LivingImage v4.3.1 software by drawing regions of interests (ROIs) around specific organs to quantify the luminescent signal as relative luminescence units (RLU).

### Cell staining, flow cytometry and fluorescence-activated cell sorting (FACS)

Parasite cultures were analysed on a MACSQuant Analyzer 10 (Miltenyi Biotec) after a 10 min centrifugation at 4,105×*g* and resuspension in PBS + 0.2% BSA buffer. Analyses were performed using FlowLogic Software (Miltenyi Biotec) using a specific gating for singlet parasites expressing dsRed, for which the non-transfected parental parasite line served as a control. In some experiments, parasites were stained with 5-(and 6)-carboxyfluorescein diacetate succinimidyl ester (CFSE; Cell Division Tracker Kit, BioLegend) according to manufacturer’s instructions. Briefly, lyophilized CFSE was reconstituted with DMSO to a stock concentration of 5 mM. This stock solution was diluted in PBS to a 5 μM working solution. Promastigotes at a concentration of 10^8^ cells/mL were centrifuged at 4,000×*g* for 10 min and resuspended in CFSE working solution for 20 min at 25°C. The staining was quenched by adding 5 times the original staining volume of cell culture medium containing 10% FBS. Parasites were centrifuged again and resuspended in pre-warmed HOMEM medium for 10 min. After incubation, CFSE labeled parasites were used for infection and determining *in situ* proliferation in macrophages, LT-HSC and human HSPC.

Sorting of mouse LT-HSC and human HSPC was performed, and quality confirmed as described previously [16]. Briefly, BM cell suspensions (2×10^7^/mL concentration) were treated with FcɣR-blocking agent (anti-CD16/32, clone 2.4G2, BD Biosciences) for 15 min, followed by a washing step using 400×*g* centrifugation and resuspension in PBS + 0.2% BSA buffer. Next, cells were incubated for 20 min at 4°C with a mix of fluorescent conjugated anti-mouse antibodies at optimized concentrations. DAPI Staining Solution (Miltenyi Biotec) was used to assess viability. A 96-well plate (Greiner Bio-One) was prepared for sorting by adding RPMI 1640 medium supplemented with 1% NEAA, 100 U/mL penicillin, 100 μL streptomycin, 500 μg/mL gentamycin, 2 mM L-glutamine, 1 mM sodium pyruvate and 10% iFCS to the wells in which 10,000 LT-HSC/well were sorted using FACSMelody (BD Bioscience) following specific gating strategies, confirmed with fluorescence minus one (FMO) controls and compensated using single stains, as described and shown in S7 Fig and Tables 1–3 of our previous report [16]. For visualizing infection, LT-HSC were collected on slides by Cytospin, fixed using methanol and stained for 15 min with Giemsa (Sigma Aldrich). Microscopic images were acquired using the UltraVIEW VoX dual spinning disk confocal system (PerkinElmer). For image analysis, z-stack imaging was included and a z-projection using the max intensity method was created for each image. The region-of-interest (ROI) was then manually drawn around the DsRed^+^ signals of the amastigotes, allowing quantification of the area and fluorescence intensity using the FIJI software. For analysis of dsRed and/or CFSE levels on amastigotes at designated timepoints, infected macrophages and LT-HSC were recovered from the cultures. Cells were centrifuged at 400×*g* for 10 min and in PBS + 0.2% BSA. Host cell membranes were disrupted by 3 passages through a 25G needle. Amastigotes were collected in the supernatant after centrifugation at 250×*g* for 10 min and subsequently pelleted at 3,000×*g* for 10 min and resuspended in 500 μL PBS + 0.2% BSA for analysis by flow cytometry.

### RNA isolation

Total RNA was extracted from three independent samples of 10,000 FACSMelody-sorted DsRed^hi^ amastigotes or quiescent amastigotes (*L. infantum* LEM3323^PpyRE9/DsRed^) obtained from 5,000 sorted and infected LT-HSC. Extraction was performed with the QIAamp RNA Blood Mini kit (Qiagen), according to the manufacturer’s instructions. To exclude gDNA, an additional step using gDNA elimination columns (Monarch) was performed. RNA samples were stored in aliquots at -80°C.

### RT-Digital PCR (RT-dPCR)

RT-dPCR was conducted using a QIAcuity dPCR system (QIAGEN), employing nanoplate PCR plates (8.5 k partitions, 96 well) and the QIAcuity OneStep Advanced EG Kit (cycling conditions: reverse transcription at 50°C for 40 minutes, initial denaturation at 95.0°C for 2 minutes, followed by 40 cycles of denaturation at 95.0°C for 10 seconds, annealing/extension at 60.0°C for 30 seconds, and a final extension at 40.0°C for 5 minutes. Imaging was with an exposure duration of 500 ms and gain set to 6. Duplicate assays were performed for 18 different genes (**S2 Table**) on RNA extracts from sorted DsRed^hi^ and DsRed^lo^ amastigotes. From the obtained data, extracted RNA copy numbers per 10^4^ parasites were calculated.

### RNA sequencing and bioinformatics

Unbiased total RNA sequencing was performed at Brightcore using the SMARTer Stranded RNA-Seq Kit to generate strand-specific RNAseq libraries for Illumina sequencing. Reads were generated in an S4 run (2×100bp, 200M reads) on an Illumina NovaSeq 6000 apparatus. Alignments of the low input RNAseqs against the *L. infantum* JPCM5 reference genome were made using BWA (Burrows-Wheeler Aligner) v0.7.15 [58] and read count was made by HTseq-count v0.12.4 [59] and normalization was performed in DESeq2 using the variance stabilizing transformation (VST) in the default unsupervised mode [60]. Differential expression analysis was largely based on a workflow using Bioconductor packages in R [61]. Euclidean distance between samples was calculated using the R function *dist* and with the Poisson Distance package *PoiClaClu* (https://CRAN.R-project.org/package=PoiClaClu) and visualized in a heatmap using pheatmap (https://CRAN.R-project.org/package=pheatmap) and the colorRampPalette function from the RColorBrewer (https://CRAN.Rproject.org/package=RColorBrewer). PCA were generated using *ggplot2* (https://CRAN.Rproject.org/package=ggplot2) and *ggrepel* (https://cran.r-project.org/web/packages/ggrepel/). The number of counts for quiescent amastigotes were three orders of magnitude lower than those of DsRed^hi^ amastigotes based on BWA alignments. Kraken2 [62] was employed for taxonomic identification of reads using the databases *plu_pf* (2023.6), *EupathDB* (2023.6), *nt_DB* (2023.6) and *standard plus Refeq protozoa & fungi* (2023.6). Alignments were inspected via IGV, indicating that many reads were only partially mapped (less than 30% of bases). These partially mapped reads did not map to *Leishmania* RNA based on Blast [63]. To eliminate over-abundant stochastically mapped reads from the alignment, conservative filtering conditions were implemented, *i*.*e*. mapping quality greater than 60 and mapped read length longer than 90 bases. For example, this was accomplished with the command line: samtools view -h -q 60 RNA-Quiscentamastigotes-Linfantum_S114.BW.bam | grep "^@\|10[1]M\|9[0–9]M" | samtools view -Sb ‐ > RNA-Quiscentamastigotes-Linfantum_S114.BW.90map.bam. Read count data were generated with htseq-count for these new BAM files with stringent conditions.

### Sand fly infections and evaluation of parasite load

Sand fly females (*L. longipalpis*) were fed with heat-inactivated heparinized mouse blood containing 5×10^6^/mL promastigotes from log-phase cultures through a chicken skin membrane. Groups were randomized by an independent researcher until data analysis to avoid bias. Blood-fed females were separated 24 h after feeding, kept in the same conditions as the colony and dissected on 5, 7, 9, and 12 days post blood meal to microscopically check the presence of parasites. Following disruption of the total gut in 50 μL PBS, the parasite load was quantified microscopically using a KOVA counting chamber [64,65]. Parasites isolated from sand flies on day 12 post blood meal were cultured at 25°C in HOMEM promastigote medium supplemented with 5% penicillin-streptomycin and checked daily for growth, to obtain post-sand fly cultures. Parasites were then transferred to a T25 flask in parallel and passaged once before measuring DsRed expression or before infection studies (*vide supra*). The latter was carried out for all conditions synchronously, using stationary phase promastigote cultures, to normalize infection experiments.

### Promastigote growth

Promastigote growth curves were made as described before [66] to compare the *in vitro* growth of quiescent *vs* non-quiescent strains. After passage through fine needles (21G and 25G) to break clustering, the promastigotes were diluted in PBS and counted by KOVA counting chamber. Exactly 5×10^5^ log-phase promastigotes/mL were seeded in 5 mL HOMEM and their number was determined by microscopic counting every 24 h for a total of 10 days. Three independent repeats of each strain were run in parallel.

### Statistics and reproducibility

Statistical analyses were performed using GraphPad Prism version 9.0.1. Tests were considered statistically significant if *p* <0.05. Growth curves were statistically compared using Wilcoxon matched-pairs signed rank test. Parasite load in sand fly infections were tested using Unpaired *t* test. Infectivity in macrophages was tested using Ordinary one-way ANOVA. MFI of DsRed expression was compared using Mann-Whitney test.

## Supporting information

S1 Table Infection of mouse peritoneal macrophages with parasites recovered from *L.infantum* LEM3323 infected or relapse BM, or purified from infected LT-HSC, either or not passaged through the sand fly vector (pre- and post-SF). (DOCX)

S2 Table Targeted genes and primer sequences used for transcriptional profiling of DsRed^hi^ and quiescent amastigotes using RT-dPCR. (DOCX)

S1 Data Transcriptional changes in quiescent amastigotes determined by RNAseq. The data file includes the read length mapping results (MAPQ_Read-length), the high quality mapping read counts (MAPQ60_RLEN90) and the GO term enrichment results for transcripts detected in quiescent parasites (GO enrichment molecular function (MF), biological processes (BP), and cellular components (CC)).
(XLSX)

S1 Fig A proportion of quiescent parasites lose the *dsRed* gene. Human HSPC were infected for 24 hours with *L. infantum* (LLM1246 WT^PpyRE9/DsRed^), amastigotes were recovered and single cell sorted for promastigote back-transformation. **(a)** Expanded monoclonal promastigote cultures (Quiescent/DsRed^+^ and Quiescent/DsRed^-^) were measured by flow cytometry. **(b, left)** qPCR on genomic DNA samples and **(b, right)** RT-qPCR on RNA samples extracted from the monoclonal Quiescent/DsRed^+^ and Quiescent/DsRed^-^ promastigote cultures.
(TIF)

S2 Fig Quiescent amastigotes exhibit a significantly reduced size compared to DsRed^hi^ amastigotes. **(a)** Histograms of FSC measurements of quiescent amastigotes (blue) and DsRed^hi^ amastigotes (red) for the different *Leishmania* strains and species used for the mouse stem cell infections. **(b)** Ratio of DsRed MFI and FSC of all quiescent and DsRed^hi^ remeasured amastigotes. **(c-d)** Flow cytometry plots showing *L. infantum* LEM3323 promastigote cultures (c) and amastigotes purified from 24h infected LT-HSC (d). From left to right events are plotted SSC versus FSC, DsRed versus FSC to select quiescent (DsRed^lo^) and non-quiescent (DsRed^hi^) parasites, and CFSE versus FSC to rule out size differences.
(TIF)

S3 Fig Promastigote growth curves remain stable between quiescent and DsRed^hi^ strains. **(a)** *In vitro* growth curves of *L. infantum* LEM3323 promastigotes recovered from quiescent and non-quiescent (DsRed^hi^) parasites in infected mouse LT-HSC, both before (pre-SF) and after sand fly passage (post-SF). **(b)** *In vitro* promastigote growth curves of quiescent and non-quiescent (DsRed^hi^) *L. infantum* LLM2346 strains recovered from infected human HSPC. Wilcoxon matched-pairs signed rank test, **p <0.01. **(c)** *In vitro* growth curves of *L. infantum* LEM3323 promastigotes recovered from relapsed and infected BALB/c mice as described above. All results are based on three independent replicates.
(TIF)

S4 Fig RNAseq data from quiescent amastigotes recovered from mouse LT-HSC. **(a)** Principal component analysis (PCA) of the RNAseq data revealing distant clustering of the independent DsRed^hi^ and quiescent samples. **(b)** Euclidean distance matrix between the samples illustrating the Poisson Distance. **(c)** Sorted amastigotes (DsRed^hi^ and quiescent) of infected LT-HSC were RNA extracted and subjected to RT-dPCR for 18 target genes (**S2 Table**). **(d)** GO term analysis of 167 genes that are found to be expressed in the three independent quiescent *Leishmania* amastigote samples. Visual representation of GO terms enriched in biological processes, molecular function and cellular components.
(TIF)

S5 Fig BMDM were infected with *L.infantum* (LEM3323 WT^PpyRE9/DsRed^) for 24 hours followed by treatment with 250 μM PMM or 7.5 μM MIL for 72 hours. To compare pre- and post-treatment distribution of quiescent parasites, amastigotes were isolated and remeasured on the FACSMelody.
(TIF)

S6 Fig Flow cytometric measurement of stationary phase promastigote cultures. All cultures used in macrophage infection experiments were visually inspected to contain > 90% metacyclics and measured by flow cytometry to assess cellular homogeneity. Representative SSC/FSC-plots are shown for the used infection conditions. Log phase *L. infantum* LEM3323 promastigotes were included as controls.
(TIF)

## References

[1] Burza S, Croft SL, Boelaert M. Leishmaniasis. Lancet. 2018;392(10151):951–70. doi: 10.1016/S0140-6736(18)31204-2 30126638

[2] Ready PD. Epidemiology of visceral leishmaniasis. Clin Epidemiol. 2014;6:147–54. doi: 10.2147/CLEP.S44267 24833919 PMC4014360

[3] Kamhawi S. Phlebotomine sand flies and Leishmania parasites: friends or foes? Trends Parasitol. 2006;22(9):439–45. doi: 10.1016/j.pt.2006.06.012 16843727

[4] Oliveira F, de Carvalho AM, de Oliveira CI. Sand-fly saliva-leishmania-man: the trigger trio. Front Immunol. 2013;4:375. doi: 10.3389/fimmu.2013.00375 24312093 PMC3832839

[5] Feijo D, Tiburcio R, Ampuero M, Brodskyn C, Tavares N. Dendritic Cells and Leishmania Infection: Adding Layers of Complexity to a Complex Disease. J Immunol Res. 2016;2016:3967436. doi: 10.1155/2016/3967436 26904694 PMC4745329

[6] Martinez-Lopez M, Soto M, Iborra S, Sancho D. Leishmania Hijacks Myeloid Cells for Immune Escape. Front Microbiol. 2018;9:883. doi: 10.3389/fmicb.2018.00883 29867798 PMC5949370

[7] Kedzierski L, Evans KJ. Immune responses during cutaneous and visceral leishmaniasis. Parasitology. 2014:1–19. doi: 10.1017/S003118201400095X 25075460

[8] Gossage SM, Rogers ME, Bates PA. Two separate growth phases during the development of Leishmania in sand flies: implications for understanding the life cycle. Int J Parasitol. 2003;33(10):1027–34. doi: 10.1016/s0020-7519(03)00142-5 13129524 PMC2839921

[9] Horrillo L, Castro A, Matia B, Molina L, Garcia-Martinez J, Jaqueti J, et al. Clinical aspects of visceral leishmaniasis caused by L. infantum in adults. Ten years of experience of the largest outbreak in Europe: what have we learned? Parasit Vectors. 2019;12(1):359. doi: 10.1186/s13071-019-3628-z 31340851 PMC6657057

[10] Rai K, Cuypers B, Bhattarai NR, Uranw S, Berg M, Ostyn B, et al. Relapse after treatment with miltefosine for visceral leishmaniasis is associated with increased infectivity of the infecting Leishmania donovani strain. mBio. 2013;4(5):e00611–13. doi: 10.1128/mBio.00611-13 24105765 PMC3791894

[11] Tanaka N, Ashour D, Dratz E, Halonen S. Use of human induced pluripotent stem cell-derived neurons as a model for Cerebral Toxoplasmosis. Microbes Infect. 2016;18(7–8):496–504. doi: 10.1016/j.micinf.2016.03.012 27083472

[12] Ferreira-da-Silva Mda F, Takacs AC, Barbosa HS, Gross U, Luder CG. Primary skeletal muscle cells trigger spontaneous Toxoplasma gondii tachyzoite-to-bradyzoite conversion at higher rates than fibroblasts. Int J Med Microbiol. 2009;299(5):381–8. doi: 10.1016/j.ijmm.2008.10.002 19097936

[13] Ferreira AV, Segatto M, Menezes Z, Macedo AM, Gelape C, de Oliveira Andrade L, et al. Evidence for Trypanosoma cruzi in adipose tissue in human chronic Chagas disease. Microbes Infect. 2011;13(12–13):1002–5. doi: 10.1016/j.micinf.2011.06.002 21726660 PMC3552247

[14] Shanks GD, White NJ. The activation of vivax malaria hypnozoites by infectious diseases. Lancet Infect Dis. 2013;13(10):900–6. doi: 10.1016/S1473-3099(13)70095-1 23809889

[15] Beamer G, Major S, Das B, Campos-Neto A. Bone marrow mesenchymal stem cells provide an antibiotic-protective niche for persistent viable Mycobacterium tuberculosis that survive antibiotic treatment. Am J Pathol. 2014;184(12):3170–5. doi: 10.1016/j.ajpath.2014.08.024 25451154 PMC4261085

[16] Dirkx L, Hendrickx S, Merlot M, Bulte D, Starick M, Elst J, et al. Long-term hematopoietic stem cells as a parasite niche during treatment failure in visceral leishmaniasis. Commun Biol. 2022;5(1):626. doi: 10.1038/s42003-022-03591-7 35752645 PMC9233693

[17] Karagiannis K, Gannavaram S, Verma C, Pacheco-Fernandez T, Bhattacharya P, Nakhasi HL, et al. Dual-scRNA-seq analysis reveals rare and uncommon parasitized cell populations in chronic L. donovani infection. Cell Rep. 2023;42(9):113097. doi: 10.1016/j.celrep.2023.113097 37682713

[18] Cabral DJ, Wurster JI, Belenky P. Antibiotic Persistence as a Metabolic Adaptation: Stress, Metabolism, the Host, and New Directions. Pharmaceuticals (Basel). 2018;11(1). doi: 10.3390/ph11010014 29389876 PMC5874710

[19] Lewis K. Persister cells. Annu Rev Microbiol. 2010;64:357–72. doi: 10.1146/annurev.micro.112408.134306 20528688

[20] Lewis K. Persister cells, dormancy and infectious disease. Nat Rev Microbiol. 2007;5(1):48–56. doi: 10.1038/nrmicro1557 17143318

[21] Rittershaus ES, Baek SH, Sassetti CM. The normalcy of dormancy: common themes in microbial quiescence. Cell Host Microbe. 2013;13(6):643–51. doi: 10.1016/j.chom.2013.05.012 23768489 PMC3743100

[22] Ehrt S, Schnappinger D, Rhee KY. Metabolic principles of persistence and pathogenicity in Mycobacterium tuberculosis. Nat Rev Microbiol. 2018;16(8):496–507. doi: 10.1038/s41579-018-0013-4 29691481 PMC6045436

[23] Dworkin J, Harwood CS. Metabolic Reprogramming and Longevity in Quiescence. Annu Rev Microbiol. 2022;76:91–111. doi: 10.1146/annurev-micro-041320-111014 35417196

[24] Jara M, Barrett M, Maes I, Regnault C, Imamura H, Domagalska MA, et al. Transcriptional Shift and Metabolic Adaptations during Leishmania Quiescence Using Stationary Phase and Drug Pressure as Models. Microorganisms. 2022;10(1). doi: 10.3390/microorganisms10010097 35056546 PMC8781126

[25] Jara M, Maes I, Imamura H, Domagalska MA, Dujardin JC, Arevalo J. Tracking of quiescence in Leishmania by quantifying the expression of GFP in the ribosomal DNA locus. Sci Rep. 2019;9(1):18951. doi: 10.1038/s41598-019-55486-z 31831818 PMC6908629

[26] Saraiva EM, Pinto-da-Silva LH, Wanderley JL, Bonomo AC, Barcinski MA, Moreira ME. Flow cytometric assessment of Leishmania spp metacyclic differentiation: validation by morphological features and specific markers. Exp Parasitol. 2005;110(1):39–47. doi: 10.1016/j.exppara.2005.01.004 15804377

[27] Rijal S, Ostyn B, Uranw S, Rai K, Bhattarai NR, Dorlo TP, et al. Increasing failure of miltefosine in the treatment of Kala-azar in Nepal and the potential role of parasite drug resistance, reinfection, or noncompliance. Clin Infect Dis. 2013;56(11):1530–8. doi: 10.1093/cid/cit102 23425958

[28] McIvor A, Koornhof H, Kana BD. Relapse, re-infection and mixed infections in tuberculosis disease. Pathog Dis. 2017;75(3). doi: 10.1093/femspd/ftx020 28334088

[29] Nascimento TLD, Vasconcelos SP, Peres Y, Oliveira MJS, Taminato M, Souza KMJ. Prevalence of malaria relapse: systematic review with meta-analysis. Rev Lat Am Enfermagem. 2019;27:e3111. doi: 10.1590/1518-8345.2619.3111 30916225 PMC6432993

[30] Madan M, Kunal S. COVID-19 reinfection or relapse: an intriguing dilemma. Clin Rheumatol. 2020;39(11):3189. doi: 10.1007/s10067-020-05427-3 32980985 PMC7519850

[31] Barrett MP, Kyle DE, Sibley LD, Radke JB, Tarleton RL. Protozoan persister-like cells and drug treatment failure. Nat Rev Microbiol. 2019;17(10):607–20. doi: 10.1038/s41579-019-0238-x 31444481 PMC7024564

[32] Zhang Y. Persisters, persistent infections and the Yin-Yang model. Emerg Microbes Infect. 2014;3(1):e3. doi: 10.1038/emi.2014.3 26038493 PMC3913823

[33] Sanchez-Valdez FJ, Padilla A, Wang W, Orr D, Tarleton RL. Spontaneous dormancy protects Trypanosoma cruzi during extended drug exposure. Elife. 2018;7. doi: 10.7554/eLife.34039 29578409 PMC5906098

[34] Sarkar A, Saha P, Mandal G, Mukhopadhyay D, Roy S, Singh SK, et al. Monitoring of intracellular nitric oxide in leishmaniasis: its applicability in patients with visceral leishmaniasis. Cytometry A. 2011;79(1):35–45. doi: 10.1002/cyto.a.21001 21182181

[35] Voorberg-van der Wel A, Zeeman AM, van Amsterdam SM, van den Berg A, Klooster EJ, Iwanaga S, et al. Transgenic fluorescent Plasmodium cynomolgi liver stages enable live imaging and purification of Malaria hypnozoite-forms. PLoS One. 2013;8(1):e54888. doi: 10.1371/journal.pone.0054888 23359816 PMC3554669

[36] Deb C, Lee CM, Dubey VS, Daniel J, Abomoelak B, Sirakova TD, et al. A novel in vitro multiple-stress dormancy model for Mycobacterium tuberculosis generates a lipid-loaded, drug-tolerant, dormant pathogen. PLoS One. 2009;4(6):e6077. doi: 10.1371/journal.pone.0006077 19562030 PMC2698117

[37] Kloehn J, Saunders EC, O’Callaghan S, Dagley MJ, McConville MJ. Characterization of metabolically quiescent Leishmania parasites in murine lesions using heavy water labeling. PLoS Pathog. 2015;11(2):e1004683. doi: 10.1371/journal.ppat.1004683 25714830 PMC4340956

[38] Kloehn J, Boughton BA, Saunders EC, O’Callaghan S, Binger KJ, McConville MJ. Identification of Metabolically Quiescent Leishmania mexicana Parasites in Peripheral and Cured Dermal Granulomas Using Stable Isotope Tracing Imaging Mass Spectrometry. mBio. 2021;12(2). doi: 10.1128/mBio.00129-21 33824211 PMC8092208

[39] Mandell MA, Beverley SM. Continual renewal and replication of persistent Leishmania major parasites in concomitantly immune hosts. Proc Natl Acad Sci U S A. 2017;114(5):E801–E10. doi: 10.1073/pnas.1619265114 28096392 PMC5293024

[40] Jara M, Berg M, Caljon G, de Muylder G, Cuypers B, Castillo D, et al. Macromolecular biosynthetic parameters and metabolic profile in different life stages of Leishmania braziliensis: Amastigotes as a functionally less active stage. PLoS One. 2017;12(7):e0180532. doi: 10.1371/journal.pone.0180532 28742826 PMC5526552

[41] Dumetz F, Imamura H, Sanders M, Seblova V, Myskova J, Pescher P, et al. Modulation of Aneuploidy in Leishmania donovani during Adaptation to Different In Vitro and In Vivo Environments and Its Impact on Gene Expression. mBio. 2017;8(3). doi: 10.1128/mBio.00599-17 28536289 PMC5442457

[42] Shapira M, Pinelli E. Heat-Shock Protein-83 of Leishmania-Mexicana-Amazonensis Is an Abundant Cytoplasmic Protein with a Tandemly Repeated Genomic Arrangement. Eur J Biochem. 1989;185(2):231–6. doi: 10.1111/j.1432-1033.1989.tb15107.x 2684665

[43] Shrivastava R, Drory-Retwitzer M, Shapira M. Nutritional stress targets LeishIF4E-3 to storage granules that contain RNA and ribosome components in Leishmania. PLoS Negl Trop Dis. 2019;13(3):e0007237. doi: 10.1371/journal.pntd.0007237 30870425 PMC6435199

[44] Shrivastava R, Tupperwar N, Schwartz B, Baron N, Shapira M. LeishIF4E-5 Is a Promastigote-Specific Cap-Binding Protein in Leishmania. Int J Mol Sci. 2021;22(8). doi: 10.3390/ijms22083979 33921489 PMC8069130

[45] Honeyborne I, McHugh TD, Kuittinen I, Cichonska A, Evangelopoulos D, Ronacher K, et al. Profiling persistent tubercule bacilli from patient sputa during therapy predicts early drug efficacy. BMC Med. 2016;14:68. doi: 10.1186/s12916-016-0609-3 27055815 PMC4825072

[46] De Virgilio C. The essence of yeast quiescence. FEMS Microbiol Rev. 2012;36(2):306–39. doi: 10.1111/j.1574-6976.2011.00287.x 21658086

[47] Karamysheva ZN, Gutierrez Guarnizo SA, Karamyshev AL. Regulation of Translation in the Protozoan Parasite Leishmania. Int J Mol Sci. 2020;21(8). doi: 10.3390/ijms21082981 32340274 PMC7215931

[48] Reis-Cunha JL, Valdivia HO, Bartholomeu DC. Gene and Chromosomal Copy Number Variations as an Adaptive Mechanism Towards a Parasitic Lifestyle in Trypanosomatids. Curr Genomics. 2018;19(2):87–97. doi: 10.2174/1389202918666170911161311 29491737 PMC5814966

[49] Downing T, Imamura H, Decuypere S, Clark TG, Coombs GH, Cotton JA, et al. Whole genome sequencing of multiple Leishmania donovani clinical isolates provides insights into population structure and mechanisms of drug resistance. Genome Res. 2011;21(12):2143–56. doi: 10.1101/gr.123430.111 22038251 PMC3227103

[50] Ouakad M, Vanaerschot M, Rijal S, Sundar S, Speybroeck N, Kestens L, et al. Increased metacyclogenesis of antimony-resistant Leishmania donovani clinical lines. Parasitology. 2011;138(11):1392–9. doi: 10.1017/S0031182011001120 21819638

[51] Vanaerschot M, Maes I, Ouakad M, Adaui V, Maes L, De Doncker S, et al. Linking in vitro and in vivo survival of clinical Leishmania donovani strains. PLoS One. 2010;5(8):e12211. doi: 10.1371/journal.pone.0012211 20808916 PMC2923181

[52] Bulte D, Van Bockstal L, Dirkx L, Van den Kerkhof M, De Trez C, Timmermans JP, et al. Miltefosine enhances infectivity of a miltefosine-resistant Leishmania infantum strain by attenuating its innate immune recognition. PLoS Negl Trop Dis. 2021;15(7):e0009622. doi: 10.1371/journal.pntd.0009622 34292975 PMC8330912

[53] Hendrickx S, Bulte D, Mabille D, Mols R, Claes M, Ilbeigi K, et al. Comparison of Bioluminescent Substrates in Natural Infection Models of Neglected Parasitic Diseases. Int J Mol Sci. 2022;23(24). doi: 10.3390/ijms232416074 36555716 PMC9781651

[54] Volf P, Volfova V. Establishment and maintenance of sand fly colonies. J Vector Ecol. 2011;36 Suppl 1:S1–9. doi: 10.1111/j.1948-7134.2011.00106.x 21366760

[55] Lo Celso C, Scadden D. Isolation and transplantation of hematopoietic stem cells (HSCs). J Vis Exp. 2007(2):157. doi: 10.3791/157 18830434 PMC2532946

[56] Amend SR, Valkenburg KC, Pienta KJ. Murine Hind Limb Long Bone Dissection and Bone Marrow Isolation. J Vis Exp. 2016(110). doi: 10.3791/53936 27168390 PMC4941920

[57] Dobson KR, Reading L, Haberey M, Marine X, Scutt A. Centrifugal isolation of bone marrow from bone: an improved method for the recovery and quantitation of bone marrow osteoprogenitor cells from rat tibiae and femurae. Calcif Tissue Int. 1999;65(5):411–3. doi: 10.1007/s002239900723 10541770

[58] Li H. Aligning sequence reads, clone sequences and assembly contigs with BWA-MEM. ArXiv. 2013;1303.

[59] Anders S, Pyl PT, Huber W. HTSeq—a Python framework to work with high-throughput sequencing data. Bioinformatics. 2015;31(2):166–9. doi: 10.1093/bioinformatics/btu638 25260700 PMC4287950

[60] Love MI, Huber W, Anders S. Moderated estimation of fold change and dispersion for RNA-seq data with DESeq2. Genome Biol. 2014;15(12):550. doi: 10.1186/s13059-014-0550-8 25516281 PMC4302049

[61] Love MI, Anders S, Kim V, Huber W. RNA-Seq workflow: gene-level exploratory analysis and differential expression. F1000Res. 2015;4:1070. doi: 10.12688/f1000research.7035.1 26674615 PMC4670015

[62] Lu J, Rincon N, Wood DE, Breitwieser FP, Pockrandt C, Langmead B, et al. Metagenome analysis using the Kraken software suite. Nat Protoc. 2022;17(12):2815–39. doi: 10.1038/s41596-022-00738-y 36171387 PMC9725748

[63] Camacho C, Coulouris G, Avagyan V, Ma N, Papadopoulos J, Bealer K, et al. BLAST+: architecture and applications. BMC Bioinformatics. 2009;10:421. doi: 10.1186/1471-2105-10-421 20003500 PMC2803857

[64] Hendrickx S, Van Bockstal L, Bulte D, Mondelaers A, Aslan H, Rivas L, et al. Phenotypic adaptations of Leishmania donovani to recurrent miltefosine exposure and impact on sand fly infection. Parasit Vectors. 2020;13(1):96. doi: 10.1186/s13071-020-3972-z 32087758 PMC7036194

[65] Van Bockstal L, Sadlova J, Suau HA, Hendrickx S, Meneses C, Kamhawi S, et al. Impaired development of a miltefosine-resistant Leishmania infantum strain in the sand fly vectors Phlebotomus perniciosus and Lutzomyia longipalpis. Int J Parasitol Drugs Drug Resist. 2019;11:1–7. doi: 10.1016/j.ijpddr.2019.09.003 31525614 PMC6804374

[66] Hendrickx S, Leemans A, Mondelaers A, Rijal S, Khanal B, Dujardin JC, et al. Comparative Fitness of a Parent Leishmania donovani Clinical Isolate and Its Experimentally Derived Paromomycin-Resistant Strain. PLoS One. 2015;10(10):e0140139. doi: 10.1371/journal.pone.0140139 26469696 PMC4607421

