# Supplementary material for: Long-term hematopoietic stem cells trigger quiescence in Leishmania parasites
Source: PLoS Pathog. 2024 Apr 24;20(4):e1012181. doi: 10.1371/journal.ppat.1012181 (PMC11073788; doi:10.1371/journal.ppat.1012181)
Supplement: S1 Table — (DOCX) [file ppat.1012181.s001.docx]

**S1 Table.** Infection of mouse peritoneal macrophages with parasites recovered from L. infantum LEM3323 infected or relapse BM, or purified from infected LT-HSC, either or not passaged through the sand fly vector (pre- and post-SF).

|  | % infected cells | Number of parasites per 100 macrophages |
| --- | --- | --- |
| Control | 92% | 277 |
| Infection | 91% | 1166 |
| Relapse | 100% | 1751 |
| DsRed^hi^ pre-SF | 97% | 1087 |
| DsRed^hi^ post-SF | 98% | 949 |
| Quiescent pre-SF | 88% | 1606 |
| Quiescent post-SF | 95% | 2364 |
