# Supplementary material for: Long-term hematopoietic stem cells trigger quiescence in Leishmania parasites
Source: PLoS Pathog. 2024 Apr 24;20(4):e1012181. doi: 10.1371/journal.ppat.1012181 (PMC11073788; doi:10.1371/journal.ppat.1012181)
Supplement: S2 Table — (DOCX) [file ppat.1012181.s002.docx]

**S2 Table.** Targeted genes and primer sequences used for transcriptional profiling of DsRed^hi^ and quiescent amastigotes using RT-dPCR.

| **Gene ID** | **Annotated function** | **Forward primer (5'-3')** | **Reverse primer (5'-3')** | **Amplicon length** |
| --- | --- | --- | --- | --- |
| LINF_050008500 | Trypanothione reductase | GAACCCGACGAAGATCACGA | ACCGTTCTTTCCTGTTCGGA | 163 |
| LINF_360010100 | Hypothetical protein | GTACTGTGCTTTGGTAACTGCG | TCATCGGTCCAATCAGGGTG | 155 |
| LINF_350050500 | CS domain containing protein – putative | ACCAAGGCCACTAAGAGCTG | TCCATATCACCCATACCGCC | 167 |
| LINF_100016800 | Histone H3 – putative | TCGAAGAAGAGCAAGAAGGC | GATCAGCAGGCTCGTACTCT | 126 |
| LINF_140012700 | Fatty acid elongase - putative | CGTCACCCTCCCTCTCAAAT | CAATCAGGAGCGGAATGTGG | 114 |
| LINF_350044100 | Nucleoside diphosphate kinase - putative | TTGCCGGAGTACAAGAAGCT | GAGGGAAAAGAACGTGGCAC | 142 |
| LINF_330022900 | RNA recognition motif – putative | TACAGTAGCGCTATGGGGTG | GCTAATCACCGAAGTCAGCG | 84 |
| LINF_020008400 | EamA-like transporter | ATCGTTGCTGGGCTGATTTC | TTCTTTGGAGTCGCTGGTCT | 81 |
| LINF_320025400 | Hypothetical protein | CGACTAGAGATGCCGCTTCT | CGAAACTGACGAAGGCCTC | 169 |
| LINF_300023000 | Flagellar-associated PapD-like/Zeta toxin - putative | TACCAAGGAGCAGAGTGTGG | CTCGAGGTTGCATGCACTAC | 95 |
| LINF_mito0010 | 12S rRNA | GGCAAGTCCTACTCTCCTTTAC | TGCTTGTTAACCTGCTCGAAC | 137 |
| LINF_mito0030 | ND8 | AGAGAGGTTTCGGGCATCAG | CTCTGGGGCAAAATTTCGCT | 70 |
| LINF_240027300 | 3-hydroxy-3-methylglutaryl-CoA synthase - putative | ACACCTGTCTTAACCTGGCG | AGCTGTTACCCGTCCACTTG | 99 |
| LINF_mito0150 | CO1 | TCCAATTAAATTTCCGCTCTCGT | GAAGGGGAAGAAGAGAGGGG | 74 |
| LINF_310038500 | Acetyl-CoA carboxylase - putative | ACTCGCCTCTCATCCTGACT | GCACCATAATCTGCTTGCCG | 102 |
| LINF_mito0190 | RPS12 | TTTTGGGTTGCAGGACGTAA | ACGACGTCAACATTAACAAACA | 164 |
| LINF_360026000 | Inosine-guanosine transporter | CGTTACATTCGTGCTGCTCG | TTATTCCAGAACAGCGGCGT | 141 |
| LINF_350027500 | Kinetoplastid membrane protein-11 | TCGAGCGCATGATCAAGGAA | TCTGCTTGAAGTGCTCCGAG | 75 |
