# Supplementary figures and images for: Long-term hematopoietic stem cells trigger quiescence in Leishmania parasites
Source: PLoS Pathog. 2024 Apr 24;20(4):e1012181. doi: 10.1371/journal.ppat.1012181 (PMC11073788; doi:10.1371/journal.ppat.1012181)

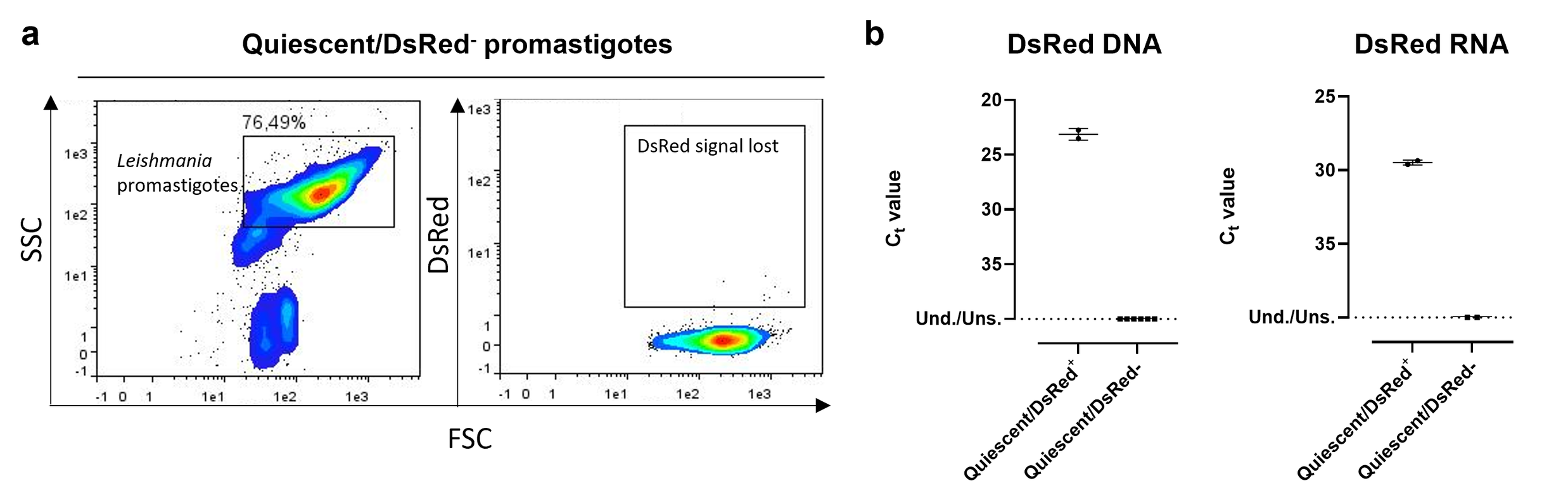

Supplement: S1 Fig — Human HSPC were infected for 24 hours with L. infantum (LLM1246 WTPpyRE9/DsRed), amastigotes were recovered and single cell sorted for promastigote back-transformation. (a) Expanded monoclonal promastigote cultures (Quiescent/DsRed+ and Quiescent/DsRed-) were measured by flow cytometry. (b, left) qPCR on genomic DNA samples and (b, right) RT-qPCR on RNA samples extracted from the monoclonal Quiescent/DsRed+ and Quiescent/DsRed- promastigote cultures. (TIF) [file ppat.1012181.s004.tif]

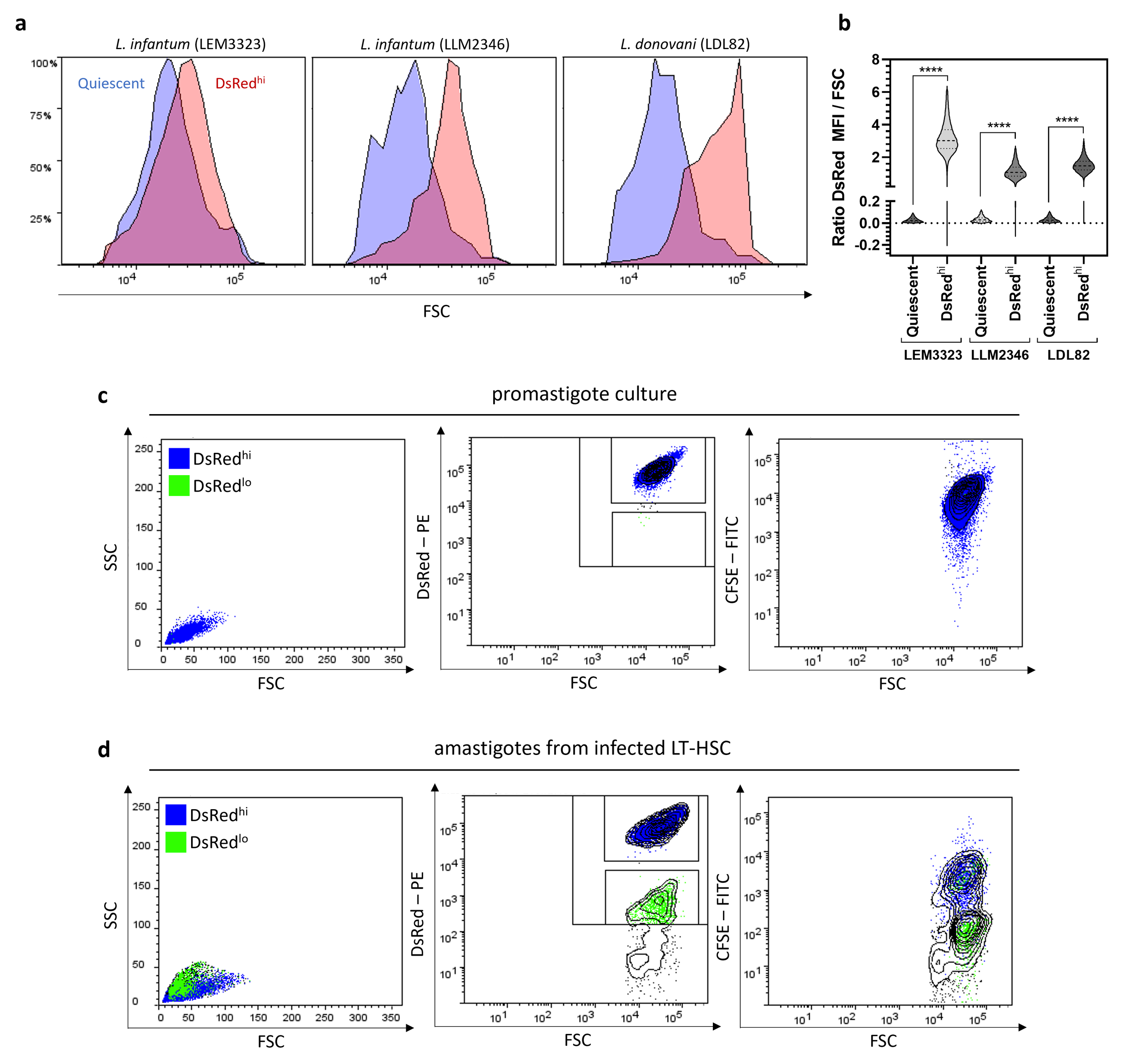

Supplement: S2 Fig — (a) Histograms of FSC measurements of quiescent amastigotes (blue) and DsRedhi amastigotes (red) for the different Leishmania strains and species used for the mouse stem cell infections. (b) Ratio of DsRed MFI and FSC of all quiescent and DsRedhi remeasured amastigotes. (c-d) Flow cytometry plots showing L. infantum LEM3323 promastigote cultures (c) and amastigotes purified from 24h infected LT-HSC (d). From left to right events are plotted SSC versus FSC, DsRed versus FSC to select quiescent (DsRedlo) and non-quiescent (DsRedhi) parasites, and CFSE versus FSC to rule out size differences. (TIF) [file ppat.1012181.s005.tif]

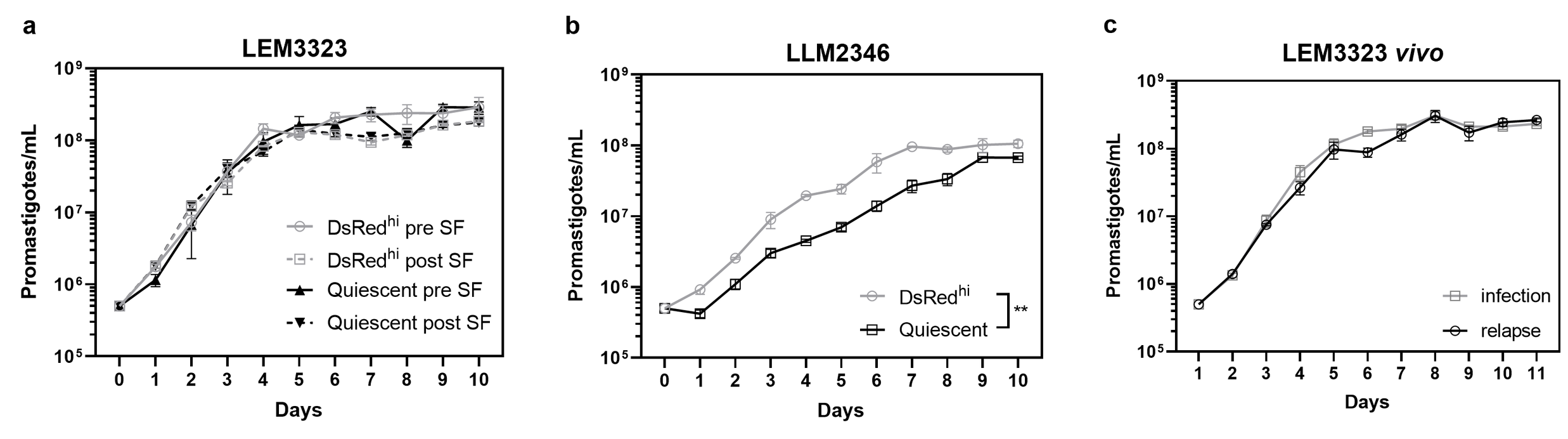

Supplement: S3 Fig — (a) In vitro growth curves of L. infantum LEM3323 promastigotes recovered from quiescent and non-quiescent (DsRedhi) parasites in infected mouse LT-HSC, both before (pre-SF) and after sand fly passage (post-SF). (b) In vitro promastigote growth curves of quiescent and non-quiescent (DsRedhi) L. infantum LLM2346 strains recovered from infected human HSPC. Wilcoxon matched-pairs signed rank test, **p <0.01. (c) In vitro growth curves of L. infantum LEM3323 promastigotes recovered from relapsed and infected BALB/c mice as described above. All results are based on three independent replicates. (TIF) [file ppat.1012181.s006.tif]

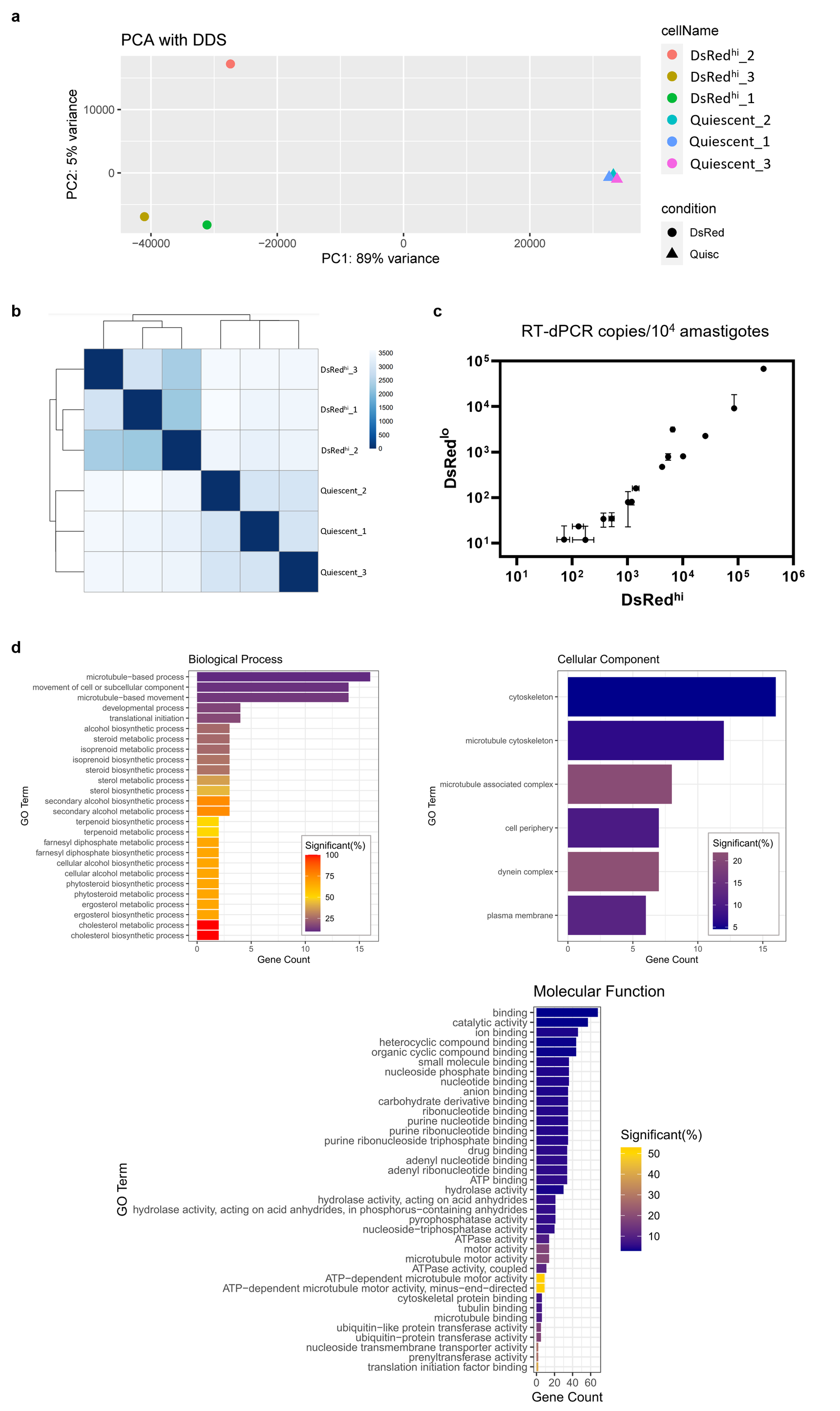

Supplement: S4 Fig — (a) Principal component analysis (PCA) of the RNAseq data revealing distant clustering of the independent DsRedhi and quiescent samples. (b) Euclidean distance matrix between the samples illustrating the Poisson Distance. (c) Sorted amastigotes (DsRedhi and quiescent) of infected LT-HSC were RNA extracted and subjected to RT-dPCR for 18 target genes (S2 Table). (d) GO term analysis of 167 genes that are found to be expressed in the three independent quiescent Leishmania amastigote samples. Visual representation of GO terms enriched in biological processes, molecular function and cellular components. (TIF) [file ppat.1012181.s007.tif]

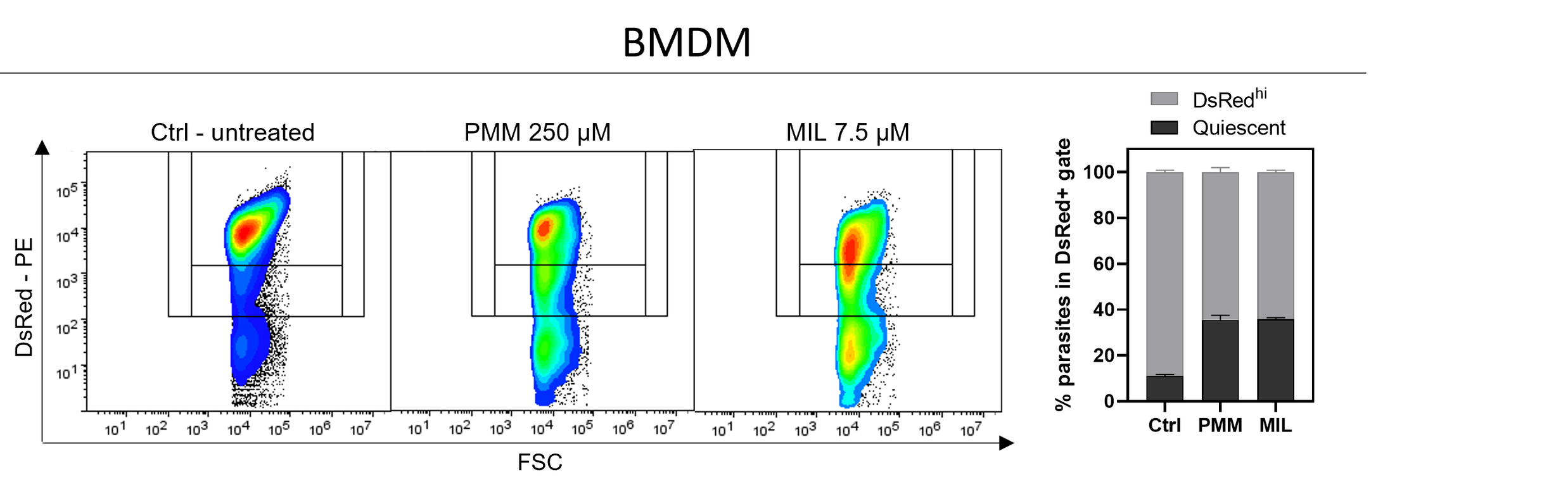

Supplement: S5 Fig — To compare pre- and post-treatment distribution of quiescent parasites, amastigotes were isolated and remeasured on the FACSMelody. (TIF) [file ppat.1012181.s008.tif]

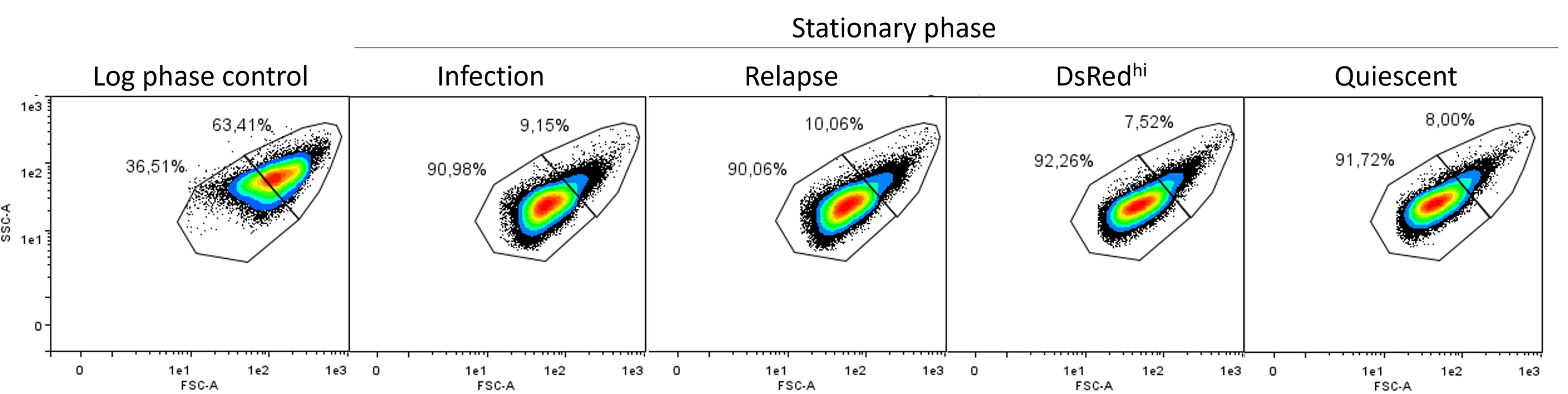

Supplement: S6 Fig — All cultures used in macrophage infection experiments were visually inspected to contain > 90% metacyclics and measured by flow cytometry to assess cellular homogeneity. Representative SSC/FSC-plots are shown for the used infection conditions. Log phase L. infantum LEM3323 promastigotes were included as controls. (TIF) [file ppat.1012181.s009.tif]
